# Prognostic factors for cognitive intervention responsiveness in Parkinson's disease: a systematic review revealing inconsistent methodology and evidence

**DOI:** 10.3389/fcogn.2026.1959713

**Published:** 2026-10-21

**Authors:** Angela Colucci, Martina Berri, Anja Ophey, Eleonora Fiorenzato, Elke Kalbe, Ann-Kristin Folkerts

**Affiliations:** 1 Department of General Psychology, University of Padua, Padua, Italy; 2 Medical Psychology, Neuropsychology and Gender Studies & Centre for Neuropsychological Diagnostics and Intervention (CeNDI), Faculty of Medicine and University Hospital Cologne, University of Cologne, Cologne, Germany; 3 Neurodegenerative Disorder Unit, Department of Neuroscience, University of Padua, Padua, Italy

**Keywords:** cognition, cognitive training, Parkinson's disease, prediction, prognostic research, systematic review

## Abstract

Cognitive dysfunction is among the most disabling non-motor symptoms experienced by people with Parkinson's disease (PwPD). Although cognitive interventions may improve cognitive functioning, reported effects are generally small and heterogeneous. Therefore, understanding how patient characteristics and intervention features influence responsiveness to cognitive interventions is essential. To date, no systematic review has specifically examined prognostic factors and models associated with responsiveness to cognitive interventions in PwPD. This systematic review aimed to provide an overview of the evidence on prognostic factors and models of responsiveness to cognitive interventions in PwPD. MEDLINE, Web of Science Core Collection, and CINAHL were searched up to June 2025. Full-text publications in English, German, or Italian were eligible for inclusion. Studies were included if they involved PwPD aged ≥18 years, irrespective of sex or cognitive status, and investigated prognostic factors and/or predictive models of responsiveness to cognitive interventions for cognitive or non-cognitive outcomes. Risk of bias was assessed using the Quality in Prognosis Studies (QUIPS) tool. Findings were synthesized narratively by extracting relevant information from the included studies. Twenty-seven publications comprising 835 PwPD were included. Across studies, eleven factor finding approaches and one factor finding model were employed. Individual-related factors (e.g., age, education, cognitive baseline level) were investigated more frequently than intervention-related factors (e.g., analog vs. digital). The most consistent finding was that lower baseline cognitive performance was associated with greater gains following cognitive intervention. Risk-of-bias assessment revealed substantial methodological limitations across studies. The conclusions that can be drawn from the current evidence are limited by considerable heterogeneity in study methodology and investigated factors. Determining which characteristics influence responsiveness to cognitive intervention in PwPD remains an important research gap.

**Systematic review registration:**

PROSPERO https://www.crd.york.ac.uk/PROSPERO/view/CRD420251079163, identifier: CRD420251079163.

## 1 Introduction

Parkinson's disease (PD) is a neurodegenerative disorder clinically characterized by specific motor symptoms, including bradykinesia, rigidity, and tremor. Among the non-motor symptoms, cognitive dysfunctions belong to the most disabling features of PD. The “cognitive continuum” in people with PD (PwPD) ranges from healthy, age-appropriate cognitive performance to subjective cognitive decline (PD-SCD), which is not detectable on objective testing, to mild cognitive impairment (PD-MCI), characterized by psychometrically verified cognitive deficits, and PD dementia (PDD), which negatively affects the PwPD's activities of daily living (Aarsland et al., 2021). Recent estimates indicate that PD-MCI affects around 40% of PwPD, while the cross-sectional prevalence of PDD ranges from 24 to 31% (Baiano et al., 2020; Aarsland et al., 2021). Typically, memory and executive functions are affected early, although impairments in attention, visuospatial abilities, and language are also common (Kalbe et al., 2016). Cognitive deficits in PwPD are of considerable clinical relevance, as they are associated with disease progression, mortality, nursing home placement, and substantial impairments in quality of life, daily functioning, social relationships, and caregiver burden (Leroi et al., 2012; Aarsland et al., 2021).

As pharmacological treatment for PDD, acetylcholinesterase inhibitors (i.e., rivastigmine) are recommended (Mamikonyan et al., 2015; Wang et al., 2015; Kalbe et al., 2024). Among non-pharmacological treatment options, cognitive interventions are gaining growing attention (Biundo et al., 2017; Kalbe et al., 2024). However, available research on non-pharmacological treatments in PD still appears fragmented and would benefit from more systematic investigation (Naeim et al., 2026), especially considering that recent evidence suggests positive effects on cognitive and partly also non-cognitive outcomes (Leung et al., 2015; Lawrence et al., 2017; Couture et al., 2019; Gavelin et al., 2022).

Cognitive interventions can be described as therapeutic approaches aiming to enhance, strengthen, or maintain cognition. According to Bahar-Fuchs et al. (2019), they can be distinguished into three categories. Cognitive training (CT) refers to standardized analog or digital exercises. Cognitive rehabilitation (CR) uses patient-tailored methods to reach identified individual intervention goals. Lastly, cognitive stimulation (CS) consists of a range of cognitive and social activities to indirectly stimulate cognitive and non-cognitive functions.

Although a Cochrane review on CT in people with PD-MCI and PDD did not find significant positive effects on cognition or non-cognitive outcomes, the authors attributed this finding to the small number of studies and high heterogeneity in methodology among trials (Orgeta et al., 2020), other meta-analyses showed a beneficial impact of CT especially in PwPD without objective cognitive impairment and in PD-MCI (Leung et al., 2015; Gavelin et al., 2022; Tang et al., 2025). These positive effects have been attributed to CT inducing neuroplasticity within the frontostriatal circuit and the hippocampal area, which represent critical areas of cognitive dysfunctions in PwPD (Guglietti et al., 2021). Similarly, CR has proved to be beneficial in ameliorating cognition in PwPD not affected by dementia (Sanchez-Luengos et al., 2021). CS seems to result in positive effects on global cognition, quality of life, and psychological and behavioral symptoms in cases of all-cause dementia (Woods et al., 2023), including PDD, although only a few studies have been conducted in this specific group (Folkerts et al., 2018).

Rather than focusing solely on which type of intervention benefits a particular target group (e.g., PD-MCI), it may also be useful to determine which individuals are most likely to benefit from a specific intervention based on their unique characteristics. Evidence shows that responsiveness to cognitive intervention may be highly heterogeneous, as discussed in a systematic review on responsiveness to working memory training in healthy individuals (e.g., Ophey et al., 2020). In this context, both individual- and intervention-related variables have been examined as potential predictors of treatment response.

Prognostic factors are specific variables that might be related to a future outcome in a defined population (Riley et al., 2019). In the context of cognitive intervention responsiveness, prognostic factors would then refer to either individual-related (i.e., demographic, clinical, psychological, neuroimaging, and biological including genetic factors) or intervention-related variables (i.e., analog vs. digital, unstructured vs. structured, multimodal vs. single-modality intervention) that are associated with changes in cognitive or non-cognitive outcomes following cognitive interventions in PwPD. Prognostic studies aim to identify either prognostic factors or models: the former assess whether one or more variables are independently associated with an outcome, whereas the latter combine multiple factors, assign weights to each one of them, and evaluate the model's predictive performance through calibration and discrimination (Moons et al., 2009) (Table 1). Focusing on how individual characteristics influence response to intervention may facilitate a shift from a one-treatment-fits-all approach toward a personalized or stratified precision medicine approach in cognitive care (Hingorani et al., 2013; Ophey et al., 2020).

**Table 1:** Definitions of the main concepts of prognostic research.

**Prognostic factor finding study:**
Aims at identifying variables which are independently linked to future outcomes.
**Prognostic factor:**
A baseline characteristic (individual or intervention-related) that may influence future outcomes, predicting how a population will respond to an intervention.
**Prognostic model:**
Combines more than one factor, weights each of them, and assesses the model's predictive performance via calibration (i.e., model's abilities to differentiate between different outcomes) and discrimination (i.e., how well the model's predicted probabilities align with observed outcomes).

To our knowledge, no systematic review or meta-analysis has yet examined prognostic research prognostic research on responsiveness to cognitive interventions in PwPD. Against this background, the aim of the present review is to identify and describe the prognostic factors investigated and the models applied to predict responsiveness to cognitive interventions in PwPD. The research question was defined according to the PICOTS system, as indicated by the Checklist for Critical Appraisal and Data Extraction for Systematic Reviews of Prediction Modeling Studies (CHARMS) (Moons et al., 2014). The population (P) included people with a clinical diagnosis of PD aged 18 years and above, of any sex and cognitive status. The intervention (I) comprised CT, CR, or CS targeting one or more cognitive domains. Comparators (C) are not applicable in prognostic research. Cognitive and non-cognitive measures (i.e., psychological, clinical, motor) were considered as outcome variables (O). Timing (T) included baseline assessment of prognostic factors and all follow-up assessments of outcome measures. The setting (S) encompassed all relevant contexts, including outpatient, inpatient, home-based, and nursing home settings.

## 2 Methods

The preregistered review protocol of this systematic review is available in PROSPERO (https://www.crd.york.ac.uk/PROSPERO/view/CRD420251079163; Supplementary File S1). Reporting follows the Preferred Reporting Items for Systematic Reviews and Meta-Analyses (PRISMA)-Protocols (PRISMA-P Group et al., 2015). This document, together with the PRISMA checklist for systematic reviews (Page et al., 2021), is provided in Supplementary Table S2.

### 2.1 Search strategy

Since prognostic research is often not directly indexed and may involve different study designs [e.g., cohort studies, *post hoc* analyses of randomized controlled trials (RCTs)] (Riley et al., 2019; Rayner et al., 2024), a broad search filter was adopted. A systematic literature search was conducted in three databases until June 3, 2025: MEDLINE, Web of Science Core Collection, and CINAHL.

Search strings were developed to identify studies investigating prognostic factors or models associated with responsiveness to cognitive interventions in PwPD. Keywords targeted three main concepts: (i) PwPD (e.g., “Parkinson's disease”, “PD”); (ii) cognition-related terms (e.g., “cognitive,” “memory,” “brain”); and (iii) intervention-related terms (e.g., “training,” “stimulation,” “rehabilitation,” “intervention,” “therapy,” “exercise,” “retraining”). Searches were performed in titles and abstracts. The complete search strategies for all databases are reported in Supplementary Table S3.

### 2.2 Study selection and data extraction

Titles and abstracts were independently screened by two reviewers (AC and MB) using Rayyan, a web- and mobile-based platform for systematic reviews (Qatar Foundation, http://rayyan.qcri.org). Full texts of potentially eligible studies were subsequently assessed for inclusion by the same reviewers. When full texts were not accessible online, the corresponding authors were contacted. Disagreements between reviewers were resolved through discussion with additional authors (A-KF, EK, and AO) until consensus was reached. Data extraction was independently performed by AC and MB according to the CHARMS checklist (Moons et al., 2014). Extracted data included study characteristics, participant demographics (e.g., age, sex, cognitive status), intervention characteristics (e.g., intervention type, setting), prognostic factors, and prognostic model analyses. Full-text research publications published in peer-reviewed journals in English, German, or Italian were included.

### 2.3 Eligibility criteria

#### 2.3.1 Study participants

Studies including PwPD aged 18 years or older, irrespective of sex, disease stage, or cognitive status, were eligible for inclusion. This included PwPD with normal cognition, PD-SCD, PD-MCI, and PDD, although explicit characterization of cognitive status was not mandatory. Studies involving individuals in prodromal stages of PD (e.g., isolated REM sleep behavior disorder) were excluded.

#### 2.3.2 Interventions

Cognitive interventions included CT, CR and CS. Interventions could be analog, digital, or mixed; either home-based or healthcare-based, individual or group-based; and supervised or unsupervised. To be included, interventions had to comprise at least two sessions and target one or more cognitive domains. In multimodal interventions [e.g., combined with transcranial magnetic stimulation (TMS), transcranial direct current stimulation (tDCS), or physical exercise], the cognitive component had to represent at least 50% of the intervention.

#### 2.3.3 Outcomes

Eligible outcomes included short- and long-term cognitive effects, encompassing both global cognition and specific cognitive domains, as well as psychological (e.g., depression) and clinical outcomes (e.g., motor symptom severity). Outcomes had to be assessed using standardized or established instruments and could be operationalized as continuous or dichotomous variables.

#### 2.3.4 Prognostic factors, prognostic models, and study designs

Any prognostic factor potentially associated with responsiveness to cognitive interventions in PwPD was considered eligible, including demographic, disease-related, baseline cognition, behavioral, biological (e.g., neuroimaging, genetic), and intervention-related variables. Intervention characteristics were considered as potential intervention-related predictors when they represented specific and clear features (e.g., structured vs. unstructured) that could be associated with patients' responsiveness to the training. Publications were excluded when the cognitive intervention was compared only to a passive control group. Therefore, the type of intervention represented the principal distinction between groups and was interpreted as a feature associated with differential responsiveness. Studies investigating prognostic models based on combinations of these variables were also included. Studies were eligible regardless of whether they reported significant intervention effects or significant associations between prognostic factors and treatment responsiveness. No restrictions were applied regarding experimental study design. Accordingly, RCTs, controlled trials, and single-arm intervention studies were all considered eligible if they included a cognitive intervention.

### 2.4 Risk of bias assessment

Risk of bias was independently assessed by two reviewers (MB and AC) using the Quality in Prognosis Studies (QUIPS) tool (Hayden et al., 2013). Disagreements were resolved through consultation with a third reviewer (AKF). The following six domains were evaluated: study participation, study attrition, prognostic factor measurement, outcome measurement, study confounding, and statistical analysis and reporting. Each domain was rated as having low, moderate, or high risk of bias according to the rating in various subitems. A detailed description of the QUIPS assessment procedure and decision rules is provided in Supplementary Table S4. The assessment framework was adapted from Ophey et al. (2020). Consistent with that review, the QUIPS tool was applied to both prognostic factor and prognostic model studies to ensure comparability across studies.

### 2.5 Narrative data synthesis

A meta-analysis was not feasible due to substantial heterogeneity in study designs, prognostic factors, models, and outcome measures. Therefore, findings were synthesized narratively by extracting and summarizing relevant information from the included studies.

### 2.6 Certainty of evidence assessment

The certainty of evidence for prognostic factors was assessed using the Grading of Recommendations Assessment Development and Evaluation (GRADE) approach adapted for prognostic research (Huguet et al., 2013). Certainty ratings were performed separately for each outcome domain, including cognitive, motor, and clinical outcomes. The following domains were evaluated: risk of bias, inconsistency, indirectness, imprecision, and publication bias. Risk of bias was mainly judged considering the exploratory nature of prognostic analyses, methodological limitations, and variability in statistical methods across studies. Inconsistency was evaluated according to the heterogeneity in predictor effects, intervention characteristics, and outcome measures. Indirectness reflected the extent to which the included populations, prognostic factors, interventions, and outcomes were directly relevant to the review question. Imprecision was assessed by considering sample sizes and the overall robustness of the available evidence. Publication bias was evaluated qualitatively, taking into account the exploratory nature of the available literature, the predominance of small studies, and the reporting of both significant and non-significant findings. The overall certainty of evidence for each outcome was rated as high, moderate, low, or very low.

## 3 Results

### 3.1 Study flow

A total of *N* = 21,175 records were identified through our database search. After removing duplicates, titles and abstracts of *n* = 13,989 records were screened for eligibility. Given that prognostic analyses are often not specifically indexed, screening at this stage focused primarily on studies involving PD and cognitive interventions. Subsequently, *n* = 77 full-text articles were assessed for eligibility, of which *n* = 27 studies met the inclusion criteria and were included in the systematic review. Details of the study selection process and reasons for exclusion are presented in Figure 1.

**Figure 1:**
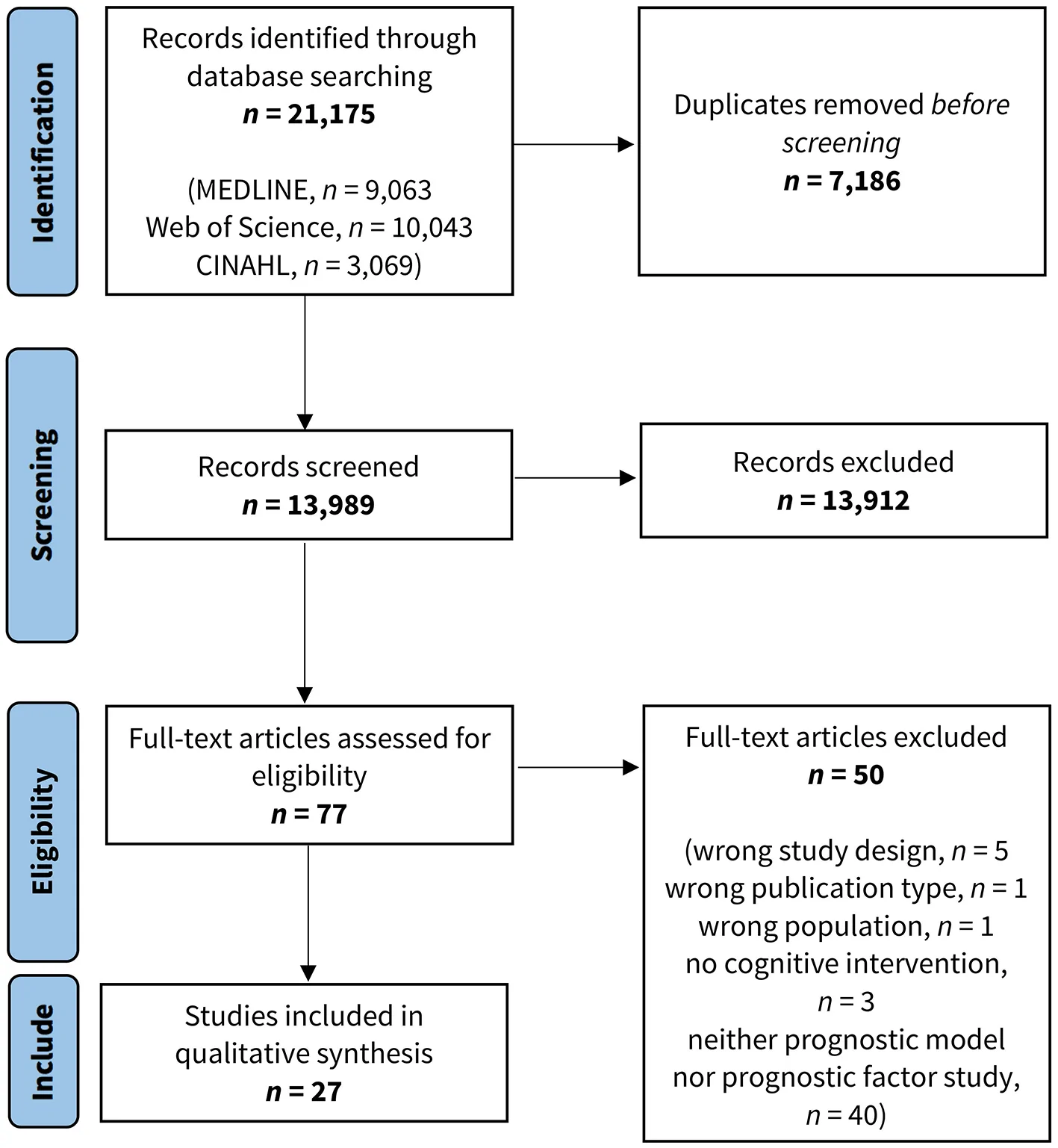
PRISMA flow diagram.

### 3.2 Sample characteristics

The 27 included publications comprised data from *n* = 983 PwPD, including *n* = 835 participants receiving a cognitive intervention and *n* = 148 controls. Mean participant age ranged from 57.8 years (Penko et al., 2019) to 71.9 years (Angelucci et al., 2015). Across studies reporting sex distribution, approximately 43% of participants were women; this information was unavailable in two studies (Costa et al., 2014; Angelucci et al., 2015). Mean educational level ranged from 5 years (Maggio et al., 2018) to 17.4 years of education (Agarwal et al., 2018; DeGutis et al., 2023), while two studies did not report educational data (Penko et al., 2019; Rosenfeldt et al., 2019). Mean disease duration ranged from 4.1 years (DeGutis et al., 2023) to 14.9 years (Bernini et al., 2021).

Reported Hoehn and Yahr (H&Y) stages ranged from 0 (Zimmermann et al., 2014) to 4 (Reuter et al., 2012; Kalbe et al., 2020; Schmidt et al., 2021), although several studies did not provide these data (Disbrow et al., 2012; Angelucci et al., 2015; Fellman et al., 2020; Bernini et al., 2021; Giehl et al., 2024). Severity of motor symptoms was assessed in the majority of cases using part III of the Unified Parkinson's Disease Rating Scale (UPDRS-III), with a few exceptions that adopted the revised and updated version, part III of the Movement Disorder Society–Unified Parkinson's Disease Rating Scale (MDS-UPDRS-III) (Milman et al., 2014; Penko et al., 2019; Rosenfeldt et al., 2019; Fiorenzato et al., 2025). Studies reporting UPDRS-III scores showed values ranging from 11.4 (DeGutis et al., 2023) to 44.6 (Maggio et al., 2018). Among those using MDS-UPDRS, values ranged from 17.6 (Milman et al., 2014) to 38.2 (Penko et al., 2019). Motor assessment data were unavailable in five publications (París et al., 2011; Disbrow et al., 2012; Edwards et al., 2013; Valdés et al., 2017; De Luca et al., 2019).

The included studies involved PwPD with normal cognition (París et al., 2011; Edwards et al., 2013; Petrelli et al., 2014; Valdés et al., 2017; Ophey et al., 2021, 2022; DeGutis et al., 2023; Giehl et al., 2024; Maggio et al., 2024), PD-SCD (Vlagsma et al., 2020), PD-MCI (París et al., 2011; Reuter et al., 2012; Costa et al., 2014; Petrelli et al., 2014, 2015; Angelucci et al., 2015; Kalbe et al., 2020; Bernini et al., 2021; Schmidt et al., 2021; Fiorenzato et al., 2025), and broadly defined “non-demented” PwPD (Maggio et al., 2018; De Luca et al., 2019; Fellman et al., 2020). No study included participants with PDD. Cognitive status was not specified in six publications (Disbrow et al., 2012; Milman et al., 2014; Zimmermann et al., 2014; Agarwal et al., 2018; Penko et al., 2019; Rosenfeldt et al., 2019). Several publications were based on the same participant samples or subsamples and investigated identical intervention protocols. Specifically, 13 publications originated from six datasets: Costa et al. (2014) and Angelucci et al. (2015); Edwards et al. (2013) and Valdés et al. (2017); Petrelli et al. (2014), 2015); Penko et al. (2019), and Rosenfeldt et al. (2019); Ophey et al. (2021, 2022) and Giehl et al. (2024); and Kalbe et al. (2020) and Schmidt et al. (2021).

Accordingly, the following sections refer to these six datasets and 14 additional independent trials rather than to each publication separately.

### 3.3 Intervention characteristics

Only four trials conducted CR as intervention (Disbrow et al., 2012; Maggio et al., 2018; De Luca et al., 2019; Vlagsma et al., 2020), while the remaining ones used CT as training regime. Among the screened publications, studies applying cognitive stimulation only investigated the direct effects of this intervention rather than potential prognostic factors associated with intervention responsiveness; therefore, they did not meet the definition of prognostic research adopted in the present review and could not be included. However, terminology describing cognitive interventions was inconsistent across several publications (París et al., 2011; Disbrow et al., 2012; Maggio et al., 2018, 2024; De Luca et al., 2019; Vlagsma et al., 2020; Bernini et al., 2021), limiting classification according to the definitions proposed by Bahar-Fuchs et al. (2019).

Total intervention duration ranged from 200 min (Agarwal et al., 2018) to 1,440 min (Maggio et al., 2018; De Luca et al., 2019), with the number of sessions varying between 10 (Disbrow et al., 2012; Agarwal et al., 2018) and 36 (Milman et al., 2014). Multidomain interventions were used in 11 trials (París et al., 2011; Reuter et al., 2012; Milman et al., 2014; Petrelli et al., 2014, 2015; Zimmermann et al., 2014; Agarwal et al., 2018; Maggio et al., 2018, 2024; De Luca et al., 2019; Penko et al., 2019; Rosenfeldt et al., 2019; Kalbe et al., 2020; Bernini et al., 2021; Schmidt et al., 2021), whereas eight trials adopted a single-domain approach. Among the latter, interventions targeted executive functioning (Disbrow et al., 2012; Costa et al., 2014; Angelucci et al., 2015; Vlagsma et al., 2020; DeGutis et al., 2023; Fiorenzato et al., 2025), working memory (Fellman et al., 2020; Ophey et al., 2021, 2022; Giehl et al., 2024), or speed of processing (Edwards et al., 2013; Valdés et al., 2017).

Six trials used analog CT (Costa et al., 2014; Petrelli et al., 2014, 2015; Angelucci et al., 2015; Penko et al., 2019; Rosenfeldt et al., 2019; Kalbe et al., 2020; Vlagsma et al., 2020; Bernini et al., 2021; Schmidt et al., 2021), whereas nine used digital platforms, including: InSight (Edwards et al., 2013; Valdés et al., 2017); Attengo^®^ (Milman et al., 2014), CogniPlus (Zimmermann et al., 2014; Vlagsma et al., 2020); Esercizi di Riabilitazione Cognitiva (ERICA) (De Luca et al., 2019); NeuroNation (Ophey et al., 2021, 2022; Giehl et al., 2024; Maggio et al., 2024); CoRE (acronym for CR) (Bernini et al., 2021); and Tonic and Phasic Alertness Training (TAPAT) (DeGutis et al., 2023).

Three more trials developed tailored interventions: motor-related digital executive functioning exercises (Disbrow et al., 2012), selected computerized games (Agarwal et al., 2018), three online working memory tasks (Fellman et al., 2020). Only two trials adopted a mixed-intervention approach including both analog and digital tasks (París et al., 2011; Reuter et al., 2012), while in two trials VR-based interventions were used (Maggio et al., 2018; Fiorenzato et al., 2025). Lastly, two trials proposed multimodal interventions, combining motor and cognitive exercises (Reuter et al., 2012; Penko et al., 2019; Rosenfeldt et al., 2019).

Detailed study and participant characteristics are summarized in Table 2, whereas additional intervention details are reported in Supplementary Table S5.

**Table 2:** Study design, participants' demographics, and clinical features.

Datasets		Study	Study design	Prognostic factors		Participant demographics					Clinical features						
		References		Individual	Intervention	Sample	Age (years)	Sex	Education (years)	Global cognition	Dementia	Cognitivestatus	H&Y range	UPDRS	LEDD	Disease duration (years)	Depression
	1	Agarwal et al., 2018	Single-arm study	•		EG: *n* = 10^§^	67.6 (5.9)	♀3♂12	17.4 (2.6)	n.a.	n.a.	n.a.	*n* = 13: 2–2.5	*n* = 13 Part III: 15: 25.9 (7.4) Total: 15: 39.2 (11.6)	602.3 (516.4)	6.6 (6.5)	*n* = 15: 1.7 (1.8)
	2a	Angelucci et al., 2015	RCT	•	•	EG: *n* = 7^§^; CG: *n* = 8^§^	EG 67.6 (10.4); CG 71.9 (6.3)	n.a.	EG 11.7 (5.6); CG 10.6 (3.9)	MMSE EG 28.3 (1.5); CG 28.1 (1.9)	Excluded	PD-MCI	n.a.	EG 27.1 (13.6); CG 24.1 (7.1)	EG 727 (319); CG 732 (338)	EG 5.7 (2.8); CG 7.9 (6.3)	BDI-II EG 7.3 (3.8); CG 8.9 (5.9)
	2b	Costa et al., 2014	Randomized double-arm study	•	•	EG: *n* = 9^§^; CG: *n* = 8^§^	EG 66.1 (7.1); CG 70.9 (4.8)	n.a.	EG1 1.2 (5.6); CG 10.6 (3.9)	MMSE EG 28.5 (1.4); CG 28.0 (1.8)	Excluded	PD-MCI	I–III	Part III: EG 25.5 (14.8); CG 19.5 (8.8)	EG 732 (258); CG 782 (345)	EG 11.0 (9.4); CG 7.2 (6.4)	Excluded BDI > 14 EG 7.8 (4.2); CG 6.6 (2.8)
	3	Bernini et al., 2021	RCT	•	•	EG1: *n* = 18^§^; EG2: *n* = 12^§^; CG: *n* = 18^§^	EG1 74.6 (5.7); EG2 69.8 (9.7); CG 69.3 (7.7)	EG1 ♀6♂12; EG2 ♀5♂7; CG ♀6♂12	EG1 9.50 (3.94); EG2 8.08 (3.63); CG 7.67 (3.50)	MMSE EG1 25.01 (2.62); EG2 25.68 (1.91); CG 25.35 (2.68) MoCA EG1 19.09 (2.84); EG2 20.83 (3.15); CG 19.17 (3.49)	Excluded	PD-MCI	n.a.	Part III: EG1 34.93 (9.3); EG2 30.9 (10.4); CG 36.5 (12.8)	n.a.	EG1 8.44 (4.30); EG2 14.88 (7.75); CG 10.67 (7.36)	BDI-II EG1 10.22 (6.00); EG2 13.83 (6.63); CG 3.20 (2.68)
ID: DRKS00010186	4a	Kalbe et al., 2020	RCT	•		EG: *n* = 33^§^; CG: *n* = 31	EG 67.7 (7.2); CG 67.5 (8.3)	EG ♀9♂24; CG ♀15♂16	EG 13.0 (2.0–20.0); CG 12.0 (9.0–20.0)	MoCA EG 25.00 (16.0–28.0); CG 25.0 (15.0–30.0)	Excluded: PDD diagnosis or impaired ADL (Pill-Questionnaire)	PD-MCI	I–IV	Part III: EG 24.0 (6.0–63.0); CG 25.0 (4.0–56.0) Part IV: EG 0 (0–9.0); CG 0.0 (0–7.0)	EG 755.5 (260.0–2,050.0); CG 715.0 (100.0–1,632.5)	(in months) EG 86.0 (10.0–361.0); CG 75.0 (18.0–174.0)	Excluded BDI-II≥20 EG 9.0 (0.0–19.0); CG 8.0 (2.0–17.0)
	4b	Schmidt et al., 2021	RCT	•		FU-1: EG: *n* = 28^§^; CG: 26 FU-2: EG: *n* = 25^§^; CG *n* = 24	FU-1: EG 67.2 (7.0); CG 67.5 (8.7) FU-2: EG 67.0 (6.6); CG 67.1 (8.9)	FU-1: EG ♀7♂21; CG ♀11♂15 FU-2: EG ♀6♂19; CG ♀10♂14	FU-1: EG 13.4 (3.8); CG 14.0 (3.3) FU-2: EG 13.2 (3.7); CG 13.9 (3.2)	MoCA FU-1: EG 25.0 (2.2); CG 24.2 (3.2) FU-2: EG 25.1 (2.1); CG 24.1 (3.3)	Excluded: PDD diagnosis or impaired ADL (Pill-Questionnaire)	PD-MCI	I–IV	Part III: FU-1: EG 25.4 (13.3); CG 25.1 (12.8) FU-2: EG 25.0 (12.3); CG 26.2 (12.7)	FU-1: EG 890.8 (519.8); CG 739.6 (411.9) FU-2: EG 935.2 (530.8); CG 739.9 (425.7)	(in months) FU-1: EG 93.1 (66.3); CG 89.5 (44.9) FU-2: EG 93.5 (68.0); CG 85.7 (44.5)	Excluded BDI-II ≥ 20 FU-1: EG 8.4 (5.7); CG 7.3 (4.1) FU-2: EG 8.3 (5.8); CG 7.6 (4.2)
	5	DeGutis et al., 2023	Quasi-experimental study	•		LPD: *n* = 9^§^; RPD: *n* = 12^§^	LPD: 65.1 (6.2); RPD: 65.9 (7.7)	LPD: ♀1♂8; RPD: ♀8♂4	LPD: 16.4 (2.1); RPD: 17.4 (2.0)	MMSE LPD: 28.2 (0.9); RPD: 28.7 (0.3)	n.a.	PD-NC	I–III	Total score: LPD: 27.7 (11.4); RPD: 22.7 (5.8) Part III: LPD: 13.8 (8.8); RPD: 11.4 (4.9)	LPD: 605.6 (255.7); RPD: 293.3 (305.0)	LPD: 4.1 (2.9); RPD: 5.7 (5.3)	BDI-II LPD: 8.2 (4.5); RPD: 6.3 (5.0)
	6	De Luca et al., 2019	RCT		•	EG: *n* = 30^§^; CG: *n* = 30^§^	EG: 61.9 (11.5); CG: 63.2 (7.3)	EG ♀14♂16; CG ♀15♂15	EG: 3.5 (0.8); CG: 3.3 (0.8)	MMSE	Included: MMSE between 11 and 24	Mild-to-moderate impairment	< III	n.a.	n.a.	n.a.	n.a.
	7	Disbrow et al., 2012	RCT	•		*n* = 30^§^	U-PD 67.4 (8.7); I-PD 69.2 (8.6)	U-PD ♀4♂12; I-PD ♀5♂9	U-PD 16.6 (2.7); I-PD 16.1 (3.2)	MMSE	Excluded: WAIS-II Digit span scaled score < 7	n.a.	n.a.	n.a.	n.a.	U-PD 4.7 (3.4); I-PD 5.6 (5.3)	BDI-II
ID: NCT01155349	8a	Edwards et al., 2013	RCT	•		EG: *n* = 32^§^; CG: 42	EG 69.38 (7.81); CG 68.17 (8.38)	EG 16♀28♂; CG 17♀26♂	EG 14.87 (2.60); CG 15.43 (2.26)	MMSE EG 28.04 (1.68); CG 28.11 1.31 MoCA EG 24.26 (3.85); CG 25.12 (2.99)	Excluded: MMSE < 24	PD-NC	I–III	n.a.	EG 684.81 (597.09); CG 684.61 (409.03)	EG 7.25 (6.14); CG 6.63 (4.89)	CES-D EG 16.81 (4.57); CG 17.56 (6.09)
	8b	Valdés et al., 2017	RCT		•	EG: *n* = 44^§^; CG: *n* = 43^§^	EG 69.38 (7.81); CG 68.17 (8.38)	EG 16♀28♂; CG 17♀26♂	EG 14.87 (2.60); CG 15.43 (2.26)	MMSE EG 28.0 (1.7); CG 28.1 1.3 MoCA EG 24.3 (3.9); CG 25.1 (3.0)	Excluded: MMSE < 24	PD-NC	I–III	n.a.	EG 684.81 (597.09); CG 684.61 (409.03)	EG 7.25 (6.14); CG 6.63 (4.89)	CES-D EG 16.81 (4.57); CG 17.56 (6.09)
	9	Fellman et al., 2020	RCT	•		EG: *n* = 26^§^; CG: *n* = 26	EG 64.8 (6.2); CG 65.5 (4.7)	EG ♀17♂9; CG ♀17♂9	Educational attainment EG 5.3 (2.1); CG 5.5 (2.3)	TICS-M EG 36.9 (3.4); CG 37.4 (2.9) TELE EG 19.4 (0.8); CG 19.8 (0.4)	Excluded: TICS-M ≤ 27; TELE < 16	Non-demented PD	n.a.	Part I: EG 2.9 (2.5); CG 3.4 (2.6) Part II: EG 9.4 (5.8); CG 9.2 (4.8)	EG 456.8 (361.8); CG 459.8 (370.4)	EG 5.2 (3.2); CG 6.0 (6.1)	Excluded GDS-30 > 20 EG 6.3 (5.9); CG 6.1 (5.0)
	10	Fiorenzato et al., 2025	RCT	•		FU-1: EG: *n* = 15^§^; CG: *n* = 15^§^ FU-2: EG: *n* = 15^§^; CG: *n* = 13^§^	EG 70.53 (6.42); CG 65.13 (9.12)	EG ♀5♂10; CG ♀5♂10	EG 11.93 (4.03); CG 11.33 (3.29)	MMSE EG 28.5 (1.6); CG 27.1 (2.5) MMP EG 29.0 (2.4); CG 26.4 (4.3)	Excluded	PD-MCI	EG 2.1 (0.57); CG 2.2 (0.46)	MDS-UPDRS-III: EG 24.9 (10.5); CG 28.7 (13.7)	EG 692.4 (386.4); CG 648.7 (307.0)	EG 8.4 (4.5); CG 7.5 (3.9)	BDI-II EG 10.33 (7.6); CG 14 (9.7)
ID: DRKS00009379	11a	Giehl et al., 2024	RCT	•		EG: *n* = 22^§^	EG 65 (9)	EG ♀10♂12	EG 15.1 (3.3)	MoCA EG 27.3 (1.8)	Excluded: Level-II diagnostic criteria for PD-MCI or PDD	PD-NC	n.a.	Part III: EG 29 (8)	EG 650 (425)	EG 6.1 (5.3)	Excluded GDS ≥ 11 EG 1.8 (1.8)
	11b	Ophey et al., 2021	RCT	•		EG: *n* = 37^§^; CG: *n* = 38	EG 64.09 (8.56); CG 63.88 (8.39)	EG ♀18♂19; CG♀17♂21	EG 15.0 (11.0–22.0); CG 15.5 (10.0–23.0)	MoCA EG 27 (24–30); CG 28 (25–30)	Excluded: Level-II diagnostic criteria for PD-MCI or PDD	PD-NC	II–III	Part III (EG&CG) 16.81 (8.28)	EG 600.00 (0–1,785); CG 590.00 (100–2,120)	EG 5.20 (0.47–22.05); CG 4.62 (0.35–27.04)	Excluded GDS ≥ 11
	11c	Ophey et al., 2022	RCT	•		EG: *n* = 37^§^	EG 64.09 (8.56)	EG ♀18♂19	EG 15.00 (11.0–22.0)	MoCA EG 27 (24–30)	Excluded: Level-II diagnostic criteria for PD-MCI or PDD	PD-NC	II–III	Part III: 28.70 (7.96)	EG 600.00 (0–1,785)	EG 5.20 (0.47–22.05)	Excluded GDS ≥ 11
	12	Maggio et al., 2018	RCT		•	EG: *n* = 10^§^; CG: *n* = 10^§^	EG: 69.9 (6.3); CG: 68.9 (10.05)	EG: ♀4♂6; CG:♀6♂4	EG: 40% PS, 20% SS, 20% HS, 20% university; CT: 10% PS, 10% SSl, 60% HS, 20% university	MMSE	Included: MMSE between 11 and 24	PD with mild-to-moderate impairment	EG 2.4 (0.5); CG 2.0 (0.4)	Part III: EG 44.6 (11.4); CG 41.6 (18.1)	EG 744.1 (266.3); CG 691.6 (262.4)	EG 9.8 (3.4); CG 8.9 (3.4)	GDS
	13	Maggio et al., 2024	RCT		•	EG1: *n* = 12^§^; EG2: *n* = 12^§^; CG: *n* = 10^§^	EG1 59.7 (9.7); EG2 63.8 (8.3); CG 66.8 (6.5)	EG1 ♀4♂8; EG2 ♀6♂6; CG ♀1♂9	EG1 13.1 (3.5); EG2 12.0 (4.1); CG 11.7 (5.2)	MMSE EG1 28.2 (1.5); EG2 28.3 (1.5); CG 27.3 (2.1) MoCA EG1 26.6 (1.8); EG2 25.1 (3.3); CG 24.0 (2.3)	Excluded	PD-NC	< 2.5	Part III: EG1 27.9 (12.7); EG2 22.1 (6.3); CG 33.8 (8.3)	EG1 373.4 (177.0); EG2 280.2 (126.1); CG 450.0 (191.5)	EG1 4.8 (2.5); EG2 5.4 (1.9); CG 3.5 (2.2)	HDRS EG1 8.0 (5.2); EG2 9.1 (5.8); CG 7.8 (6.7)
	14	Milman et al., 2014	Single-arm study	•		EG: *n* = 18^§^	EG 67.7 (6.4)	EG ♀7♂11	EG 15.5 (3.3)	MMSE EG 29.2 (1.1)	Excluded: MMSE < 24	n.a.	I–II	MDS-UPDRS-III: EG 17.6 (7.5)	n.a.	EG 8.9 (6.6)	n.a.
	15	París et al., 2011	RCT	•		EG: *n* = 16^§^; CG: *n* = 12	EG 64.8 (9.2); CG 65.4 (9.6)	EG ♀9♂7; CG ♀5♂7	EG 9.9 (2.9); CG 9.5 (3.1)	MMSE ACE	Excluded: MMSE < 23; WAIS-III < 40	PD-MC: *n* = 14; PD-NC: *n* = 14	I–III	n.a.	n.a.	EG 6.94 (4.58); CG 8.25 (9.22)	Excluded GDS-15 > 10
ID: NCT02538029	16a	Penko et al., 2019	Randomized double-arm study		•	MMT: *n* = 9^§^; SMT: *n* = 10^§^	MMT: 57.8 (8.2); SMT: 64.6 (8.5)	MMT: ♀5♂4; SMT: ♀1♂9	n.a.	n.a.	n.a.	n.a.	II–III	MDS-UPDRS-III: MMT: 34.5 (10.3); SMT: 38.2 (12.9)	MMT: 786.4 (657.6); SMT: 701.1 (537.4)	MMT: 8.1 (5.3); SMT: 5.0 (3.2)	n.a.
	16b	Rosenfeldt et al., 2019	Randomized double-arm study		•	MMT: *n* = 10^§^; SMT: *n* = 10^§^	MMT: 59 (9); SMT: 65 (8)	MMT: ♀5♂5; SMT: ♀1♂9	n.a.	n.a.	n.a.	n.a.	II–III	MDS-UPDRS-III: MMT: 35 (10); SMT: 38 (13)	MMT: 437.5 (min 405, max 916.3); SMT: 550 (min 412.5, max 756.5)	MMT: 8 (min 4, max 12); SMT: 4 (min 3, max 6)	n.a.
	17a	Petrelli et al., 2014	RCT		•	EG1: *n* = 22^§^; EG2: *n* = 22^§^; CG: *n* = 21	EG1 69.2 (4.9), 55–76; EG2 68.8 (6.7), 57–85; CG 69.1(11.6), 45–89	EG1 ♀12♂10; EG2 ♀7♂15; CG ♀9♂12	EG1 13.1 (3.8), 8–20; EG2 13.6 (3.2), 8–20; CG 12.8 (3.3), 8–20	MMSE EG1 27.9 (1.9); EG2 28.1 (1.7); CG 27.9 (2.2) DemTect EG1 13.7 (2.8); EG2 13.9 (2.6); CG 14.2 (4.2)	Excluded: MMSE < 25	PD-NC: *n* = 50; PD-MCI: *n* = 15 (EG1/EG2/CG: *n* = 5/6/4)	I–III	Part III: EG1 21.1 (7.6); EG2 19.7 (8.5); CG 20.9 (6.6)	EG1 397.4 (365.4); EG2 322.6 (155.9); CG 278.3 (170.2)	(in months) EG1 66.2 (39.5); EG2 71.3 (56.2); CG 65.0 (52.8)	BDI-II EG1 10.0 (1.7); EG2 10.6 (1.9); CG 11.1 (1.9)
LANDSCAPE	17b	Petrelli et al., 2015	RCT		•	EG1: *n* = 16^§^; EG2: *n* = 17^§^; CG: *n* = 14	EG1 69.4 (4.2); EG2 68.6 (7.3); CG 68.8 (9.2)	EG1 ♀8♂8; EG2 ♀5♂12; CG ♀5♂9	EG1 13.4 (3.9); EG2 13.1 (3.2); CG 13.4 (3.7)	MMSE EG1 27.8 (2.1); EG2 28.2 (1.8); CG 27.8 (2.5) DemTect EG1 13.8 (2.8); EG2 14.4 (2.5); CG 15.3 (2.9)	Excluded: MMSE < 25	PD-NC *n* = 25; PD-MCI: *n* = 22 (EG1/EG2/CG: *n* = 6/8/8)	I–III	Part III: EG1 21.1 (7.5); EG2 19.4 (9.4); CG 19.6 (7.6)	EG1 437.7 (402.4); EG2 245.6 (98.8); CG 278.3	n.a.	BDI-II EG1 8.6 (4.8); EG2 10.8 (5.3); CG 12.1 (12.1)
	18	Reuter et al., 2012	Randomized three-arm study		•	EG1: *n* = 71^§^; EG2: *n* = 75^§^; EG3: *n* = 76^§^	64 (4)	EG1 ♀35 ♂36; EG2 ♀36 ♂39; EG3 ♀36 ♂40	EG1 10 (1.2); EG2 11 (0.6); EG3 11 (1.0)	MMSE PANDA	Excluded	PD-MCI	II–IV	Total: EG1 59.20 (12.4); EG2 60.3 (12.4); EG3 61.5 (12.8)	n.a.	(in months) EG1 98 (8); EG2 95 (9); EG3 100 (6)	HADS-D
	19	Vlagsma et al., 2020	RCT		•	EG: *n* = 24^§^; CG: *n* = 19^§^ FU: EG *n* = 22^§^; CG: *n* = 17^§^	EG 60.21 (10.42); CG 62.58 (8.84)	EG ♀10♂14; CG ♀6♂13	EG 5.5 (1.3); CG 5.6 (1.1)	SCOPA-COG EG 28.3 (4.8); CG 28.8 (4.7)	Excluded: SCOPA-COG ≤ 17	EF SCC and/or objective impairments	I–III	Part III: EG 23.96 (9.46); CG 22.24 (11.44)	EG 727.50 (336.36); CG 652.50 (564.66)	EG 6.02 (6.26); CG 6.72 (4.97)	HADS-D EG 5.83 (3.23); CG 5.63 (3.27)
	20	Zimmermann et al., 2014	RCT	•		EG: *n* = 19^§^; CG: *n* = 20	EG 69.9 (6.3); CG 66.3 (9.7)	EG ♀6♂13; CG ♀8♂12	EG 15 (11.5, 16); CG 14 (13, 17.5)	MMSE EG 29 (28, 29.5); CG 29 (28.75, 30)	Excluded (moderate and severe dementia)	n.a.	0–2.5	Total: EG 24 (7.5, 33); CG 25 (21.75, 34)	EG 738 (300, 1,195); CG 525 (295, 930)	Age at diagnosis: EG 64.8 (8.1); CG 61.1 (11.3)	BDI-II EG 4 (2, 9.5); CG 10 (7.75, 13)

^§^Group of participants discussed in the present systematic review.

ACE, Addenbrooke's Cognitive Examination (max 100); ADL, activities of daily living; BDI-II, Beck Depression Inventory; CG, control group; CR, cognitive rehabilitation; CT, cognitive training; DemTect, Demenz-Test (max 18); dlPFC, dorso-lateral prefrontal cortex; EG, experimental group; GDS, Geriatric Depression Scale; HADS-D, Hospital Anxiety and Depression Scale; HS: high school; H&Y, Hoehn & Yahr; I-PD (impaired-PD); iVR, immersive virtual reality; LEDD, levodopa daily dose; LPD, left side of motor onset; MDS-UPDRS, Movement Disorder Society—Unified Parkinson's Disease Rating Scale; MMP, mini-mental Parkinson's test (max 32); MMSE, Mini-Mental State Examination (max 30); MMT, multimodal training group; MoCA, Montreal Cognitive Assessment (max 30); *n*, number of participants; n.a., not applicable; PANDA, Parkinson Neuropsychometric Dementia Assessment (max 30);PD, Parkinson's disease; PDD (PD dementia); PD-MCI (PD-Mild Cognitive Impairment); PD-NC, PD-normal cognition; PS, primary school; RCT, randomized controlled trial; RPD, right side of motor onset; SCOPA-COG, Scales for Outcomes in Parkinson's disease-COGnition; SMT, single-modality training group; SS: secondary school; tDCS, transcranial direct current stimulation; TELE, Telephone Screening Protocol (max 20); TICS-M, Telephone Interview for Cognitive Status-Modified (max 50); U-PD (unimpaired-PD); WAIS-II, Wechsler Adult Intelligence Scale II; WM, working memory; WMT, working memory training.

### 3.4 Predictive analyses

Statistical approaches were highly heterogeneous across studies. Twenty-six publications used a factor finding approach, including eleven different types of statistical analyses. Of note, three studies applied more than one analytical approach to investigate the role of different variables in predicting cognitive intervention responsiveness in PwPD (i.e., exploring intervention-related factors using one approach, adopting a different analysis for individual-related factors) (Disbrow et al., 2012; Bernini et al., 2021; DeGutis et al., 2023).

The most frequently used method was the general linear model, applied in twelve studies (París et al., 2011; Disbrow et al., 2012; Reuter et al., 2012; Costa et al., 2014; Petrelli et al., 2014, 2015; Angelucci et al., 2015; Penko et al., 2019; Vlagsma et al., 2020; Bernini et al., 2021; DeGutis et al., 2023; Maggio et al., 2024). Other analyses included Pearson's correlations (Edwards et al., 2013; Agarwal et al., 2018; DeGutis et al., 2023), growth models (Valdés et al., 2017; Fellman et al., 2020), ANCOVAs (De Luca et al., 2019; Maggio et al., 2024), Spearman's correlations (Bernini et al., 2021; Giehl et al., 2024), linear regressions (Disbrow et al., 2012; Zimmermann et al., 2014), multiple regression analyses (Kalbe et al., 2020; Schmidt et al., 2021), mixed-effects models (Rosenfeldt et al., 2019; Fiorenzato et al., 2025), a latent difference score model (Ophey et al., 2021), and a Wilcoxon signed-rank test (Milman et al., 2014). Most studies, using the general linear models, operationalized intervention responsiveness as change scores (i.e., posttest-pretest), whereas some used scores at different time points as dependent variables (i.e., Reuter et al., 2012; Costa et al., 2014; Angelucci et al., 2015; Vlagsma et al., 2020; DeGutis et al., 2023). For ANCOVAs, post-intervention scores were used for dependent variables. For Pearson's correlations, Spearman's correlations, latent difference score model, linear regression, mixed effects models, and multiple regressions, change scores were selected as dependent variables. Lastly, only one study developed a prediction model using random forest regression analyses with post-intervention and follow-up test scores as dependent variables (Ophey et al., 2022), performing in addition Pearson's correlations to explore directions of effects.

### 3.5 Outcome measures

Most studies focused on cognitive domains particularly vulnerable in PwPD:

- Executive functioning (París et al., 2011; Disbrow et al., 2012; Reuter et al., 2012; Costa et al., 2014; Petrelli et al., 2014; Zimmermann et al., 2014; Angelucci et al., 2015; Maggio et al., 2018, 2024; De Luca et al., 2019; Rosenfeldt et al., 2019; Kalbe et al., 2020; Vlagsma et al., 2020; Bernini et al., 2021; Ophey et al., 2021; Schmidt et al., 2021; DeGutis et al., 2023; Giehl et al., 2024; Fiorenzato et al., 2025).
- Working memory (París et al., 2011; Petrelli et al., 2014; Zimmermann et al., 2014; Rosenfeldt et al., 2019; Fellman et al., 2020; Kalbe et al., 2020; Bernini et al., 2021; Ophey et al., 2021, 2022; Schmidt et al., 2021; Giehl et al., 2024).
- Attention (París et al., 2011; Edwards et al., 2013; Petrelli et al., 2014; Zimmermann et al., 2014; Valdés et al., 2017; De Luca et al., 2019; Rosenfeldt et al., 2019; Kalbe et al., 2020; Vlagsma et al., 2020; Bernini et al., 2021; Schmidt et al., 2021; DeGutis et al., 2023; Giehl et al., 2024; Fiorenzato et al., 2025).
- Memory (París et al., 2011; Petrelli et al., 2014; Zimmermann et al., 2014; De Luca et al., 2019; Kalbe et al., 2020; Vlagsma et al., 2020; Bernini et al., 2021; Schmidt et al., 2021; DeGutis et al., 2023; Giehl et al., 2024).
- Global cognition and visuocognition (Milman et al., 2014; Petrelli et al., 2015; Agarwal et al., 2018; Penko et al., 2019; Vlagsma et al., 2020; Bernini et al., 2021).

Additional outcomes included psychological variables (e.g., depression, anxiety) (París et al., 2011; Petrelli et al., 2014; Maggio et al., 2018; De Luca et al., 2019; Kalbe et al., 2020; Schmidt et al., 2021), clinical variables (e.g., activities of daily living) (París et al., 2011; Disbrow et al., 2012; Kalbe et al., 2020; Schmidt et al., 2021; Fiorenzato et al., 2025), and motor outcomes (Disbrow et al., 2012; Reuter et al., 2012; Milman et al., 2014; Agarwal et al., 2018; Penko et al., 2019; Rosenfeldt et al., 2019; DeGutis et al., 2023). In three studies, changes in severity of motor symptoms, such as gait velocity and UPDRS-III scores, were defined as primary outcomes (Agarwal et al., 2018; Penko et al., 2019; Rosenfeldt et al., 2019). Typically, established instruments were used for the assessment of outcomes, with the exception of a few studies which used tailored exercises, for example specific individual tasks to evaluate prospective memory or motor-related executive functioning (Disbrow et al., 2012; Costa et al., 2014).

### 3.6 Predictor variables and prediction results

Included studies investigated various predictors of cognitive intervention responsiveness in PwPD, including individual-related factors, such as demographic (e.g., age, sex, education), cognitive (e.g., baseline performance, intelligence), disease-related clinical features [i.e., levodopa daily dose (LEDD), disease duration, severity of motor symptoms], neuroimaging (e.g., cortical thickness), and genetic variables [i.e., apolipoprotein E4 (ApoE4)].

Additionally, most studies explored intervention-related factors, such as unstructured vs. structured (Petrelli et al., 2014, 2015; Bernini et al., 2021), multimodal vs. single-modality (Reuter et al., 2012; Penko et al., 2019; Rosenfeldt et al., 2019), analog vs. digital (De Luca et al., 2019; Bernini et al., 2021; Maggio et al., 2024) interventions. An overview of all prognostic analyses is provided in Table 3. Further, to improve interpretability, the following sections focus on predictors examined in at least three studies.

**Table 3:** Prognostic analyses, outcomes, results, and timing.

#	Study	Prognostic factors		Analysis – type of DV	Outcome	Prognostic factor														Timing		
	References	Individual	Intervention			Age	Sex (F)	Education	Performance at T0	IQ	Depression	Brain imaging	Type of training	Dose of training	Motor severity	LEDD	Disease duration	Genetics	Others	Post-intervention	FU-1	FU-2
1	Agarwal et al., 2018	•		Pearson's corr.–ChS	Motor functioning	–	–									–	–		Time of the day the CT was conducted ↑^*^	×		
2a	Angelucci et al., 2015	•	•	Mixed ANOVA—PT	EF—Set shifting accuracy								EG ↑^*^							×		
					EF—Set shifting response time								–									
					BDNF								EG ↑^*^							×		
2b	Costa et al., 2014	•	•	ANOVA—PT	PM								EG ↑^*^							×		
					EF (phonemic fluency)/EF (semantic fluency)/EF (TMT A and B)								–									
					EF (alternate fluency)								EG ↑^*^							×		
3	Bernini et al., 2021	•	•	Kruskal–Wallis test—ChS; Spearman's correlations—ChS	Global cognition	–		–	EG1 & EG2 ↓^*^		–		EG1 > EG2 & CG^*^		–		–		IADL, cognitive reserve–	×		
					Episodic long-term memory/EF/WM								–									
					Attention/processing speed								EG1 & EG2 > CG^*^							×		
4a	Kalbe et al., 2020	•		Multiple regression analyses—ChS	Memory	–		–	↓^*^						–	–		ApoE4 ↓+		×		
					EF	↑+		↓+	↓^*^						↑^*^	↑^*^		ApoE4 ↑^*^		×		
					WM	–		–	↓^*^						–	–		–		×		
					Visuocognition	↑+		–	↓^*^						–	–		–		×		
					Language	–		–	↓^*^						↑^*^	–		ApoE4 ↑^*^		×		
					Attention/Global cognition/ADL/Self-reported physical activity/QoL/Self-experienced deficits of attention	–		–	–						–	–		–				
					Depression	↓+		–			↓^*^				–	–		–		×		
					Motor functioning	–		↑^*^							↓^*^	↓+		–		×		
4b	Schmidt et al., 2021	•		Multiple regression analyses—ChS	Memory	↓^*^	↑^*^	–	↓^*^						–	↓^*^		ApoE4 –				×12
					EF	↑^*^	–	–	↓^*^						–	–		ApoE4 –			×6	
						↓^*^	↓^*^	–	↓^*^						–	↓^*^		ApoE4 ↓^*^				×12
					Attention	–	–	↑^*^	↓^*^						–	↓^*^		ApoE4 –				×12
					WM	–	↑^*^	–	↓^*^						–	–		ApoE4 ↑^*^				×12
					Visuocognition	–	↑^*^	–	↓^*^						–	–		ApoE4 –			×6	
						–	–	–	↓^*^						–	–		ApoE4 –				×12
					Language	–	–	–	↓^*^						–	–		ApoE4 –			×6	×12
					ADL	–	–	–							–	–		ApoE4 –	ADL –			
					Self-reported physical activity	–	–	–							–	–		ApoE4 –	Self-reported physical activity ↓^*^			×12
					Depression	–	↑^*^	–			–				–	–		ApoE4 –				×12
					QoL	–	↑^*^	–							↓^*^	–		ApoE4 ↑^*^	QoL –		×6	
						–	↑^*^	–							–	–		ApoE4 –				×12
					Self-experienced attention deficits	–	–	–							–	–		ApoE4 –	Self-experienced attention deficits –			
					Motor functioning	–	–	–							↓^*^	–		ApoE4 –			×6	
5	DeGutis et al., 2023	•		ANOVA—PT; Pearson's correlations—ChS	Sustained attention/selective attention/EF/visuospatial functioning														–			
					Memory (immediate recall)														RPD ↑+	×		
					Memory (delayed recall)														–			
					Motor functioning														LPD ↑^*^	×	×1	
									T0 sustained attention ↓^*^											×		
6	De Luca et al., 2019		•	ANCOVA—PT	Global cognition/frontal abilities/fluency/anxiety/depression								–									
					Attention and orientation/memory/language/visual spatial capacity/EF								EG ↑^*^							×		
7	Disbrow et al., 2012	•		ANOVA—PT, linear Regression analysis—ChS	EC sequence completion time/ EC sequence initiation time/ Switching/ Measures involving IR motor sequencing/ADLs				–													
					IR sequence initiation time				I-PD > U-PD ^*^ TMT B –										Switching, ADL, TUG –	×		
					IR sequence completion time				I-PD > U-PD ^*^ TMT B ↑^*^										Switching, ADL, TUG –	×		
					IR sequences errors				TMT B ↑^*^										Switching, ADL, TUG –	×		
8a	Edwards et al., 2013 =^1^	•		Pearson's correlations—ChS	Speed of processing	–			Global cognition –		–				–	↑^*^	↑^*^		Age at diagnosis ↓^*^ Visual acuity – Self-rated health –	×		
8b	Valdés et al., 2017 =^1^		•	Mixed effects growth model—ChS	Speed of processing									↑^*^							×3	
9	Fellman et al., 2020	•		Growth model (GMANOVA)—ChS	WM	–		↑^*^	WM—Global cognition ↑^*^							–	↓^*^			×		
																	
10	Fiorenzato et al., 2025		•	Repeated measures linear mixed-effects models—ChS	Time-based PM/verbal-response PM								EG ↑^*^							×	×2	
					Event-based PM/action-response PM/Mist-24 (PM)/recognition PM/Attention/EF (planning, shifting, updating)/subjective perception of memory functioning/neuropsychiatric symptoms/functional status								–									
					Attention/EF (Inhibition)								EG ↑^*^							×		
11a	Giehl et al., 2024	•		Spearman's correlations—ChS	Verbal WM/non-verbal WM/visuospatial WM/verbal memory/attention/EF							Cortical thickness & white matter lesions –										
11b	Ophey et al., 2021	•		Growth model—Latent ChS	WM	↓^*^		–	↓^*^	↑^*^					–					×		
						–		↑^*^	↓^*^	–					–						×3	
					EF	–		–	–	–					–				Self-efficacy expectancy ↑+	×		
11c	Ophey et al., 2022	•		Random forest regression models—PT	Verbal WM	#	#	#	Verbal WM, non-verbal WM, EF, visuo-cognition, global cognition, other cognitive domains #	#	#				#	#			Drug therapy years, self-efficacy, baseline WM training intercepts, WM training slopes #	#	#3	
		•		Pearson's correlations—ChS		–		–	Non-verbal WM, verbal WM, EF ↑^*^, other cognitive domains –	↑^*^	–				–	–			Drug therapy years –, self-efficacy –, baseline WM training intercept ↑^*^, WM training slopes –	×		
						–		–	Non-verbal WM, verbal WM ↑^*^, other cognitive domains –	–	–				–	–			Drug therapy years –, self-efficacy –, baseline WM training intercept +, WM training slopes –		×3	
12	Maggio et al., 2018		•	ANCOVA—PT	Global cognition/EF								EG ↑^*^							×		
					Depression/anxiety								–									
13	Maggio et al., 2024		•	ANOVA/Kruskal–Wallis test—ChS	Global cognition								EG1 > CG^*^ EG2 > CG^*^							×		
					EF								EG1 > CG+ EG2 > CG^*^							×		
					Theory of mind								–									
14	Milman et al., 2014		•	Wilcoxon Signed Ranked tests—ChS	Global cognition									↑^*^							×1	
					Motor functioning									↑^*^							×1	
15	París et al., 2011	•		2-way ANOVA—ChS	Global cognition/attention and WM/processing speed/ verbal memory/learning/visual memory/QoL/visuoconstructive abilities/verbal fluency/depression/ADL cognitive difficulties				–													
					Visuospatial abilities/EF				MCI vs NC ↓^*^											×		
16a	Penko et al., 2019		•	ANOVA—ChS	Motor functioning								–									
16b	Rosenfeldt et al., 2019		•	Linear mixed effects models—ChS	More affected arm swing path length								MMT ↑^*^ (in DT)							×	×1	
					Less affected arm swing path length/gait velocity/cadence/step length/step width/WM/verbal fluency/EF								–									
					Attention								MMT ↑^*^ (in ST)							×	×1	
17a	Petrelli et al., 2014		•	ANOVA—ChS	WM								EG1 > EG2^*^							×		
					Verbal fluency/attention/verbal and visual long-term memory/visuoconstruction/QoL								–									
					Verbal short-term memory								EG1 > EG2+							×		
					Depression								EG2 > EG1+							×		
17b	Petrelli et al., 2015		•	ANOVA—ChS	Global cognition								EG1>EG2^*^								×12	
18	Reuter et al., 2012		•	Linear model for repeated measures—ChS	Global cognition/EF								EG3 > EG1 & EG2^*^							×	×6	
					QoL								EG3 > EG1 & EG2^*^								×6	
					Motor functioning								–									
19	Vlagsma et al., 2020		•	ANOVA—ChS	EF/attention/memory/QoL								–									
					Rating of goal attainment/Self-experienced EF								EG ↑^*^							×		
20	Zimmermann et al., 2014	•		Stepwise linear regression analyses—ChS	Attention/visuoconstruction/episodic memory				–													
					WM/EF				↓^*^											×		

(a)ADL, activities of daily living; ApoE4, apolipoprotein E4; BDNF, brain-derived neurotrophic factor; CG, control group; ChS, change scores; DT, dual-task; DV, dependent variable; Edu, Education; EC, externally cued; EG, experimental group; EF, executive functions; F, being female; GMANOVA, generalized multivariate analysis-of-variance; H&Y, Hoehn & Yahr scale; IADL, independent activities of daily living; Individual; Intervention; I-PD, impaired PD; IQ, intelligence; IR, internally represented; LPD, left side of motor onset; MCI, Mild Cognitive Impairment; Mist-24, Memory for Intention Screening Test; MMT, multimodal training; NC, normal cognition; PD, Parkinson's disease; PM, prospective memory; PT, post-test scores; RPD, right side of motor onset; ST, single-task; TMT A, B, Trail-Making Test A, B; TUG, timed up and go test; U-PD, unimpaired PD; UPDRS, unified PD rating scale; WM, working memory. (b) –, predictor was investigated but it not statistically significant; ↑, positive relationship between predictor and outcome; ↓, negative relationship between predictor and outcome; ^*^, relationship between predictor and outcome was statistically significant (*p* < 0.05); +, relationship between predictor and outcome showed a trend toward statistical significance; >, a type of intervention predicts greater improvements compared to another, when more than two groups were compared; #, predictor was included in the model, but the type of regression doesn't evaluate individual predictor direction and significance; statistical significance at time point of measurement, with follow-up in number of months.

### 3.7 Individual-related predictors

#### 3.7.1 Sociodemographic factors

*Age* was evaluated in six factor finding studies. Findings were inconsistent across outcomes and assessment time points. Several studies found no significant association between age and intervention responsiveness (Edwards et al., 2013; Fellman et al., 2020; Bernini et al., 2021). In contrast, Kalbe et al. (2020) reported that higher age predicted greater post-intervention improvements in executive functioning, visuocognition, and depressive symptoms. In the follow-up report of this study (Schmidt et al., 2021), higher age predicted higher responsiveness in executive functioning at 6-month follow-up, while at 12-month follow up, this relationship was reversed.

Ophey et al. (2021) found younger age to predict working memory improvements immediately after intervention, although this effect was not maintained at follow-up. Age was also identified as an important predictor in the prognostic model developed by Ophey et al. (2022), although exploratory correlations did not clarify the direction of the association.

*Education* was investigated in five factor finding studies. PwPD with higher education showed greater improvements in working memory (Fellman et al., 2020; Ophey et al., 2021), attention (Schmidt et al., 2021), and motor outcomes (Kalbe et al., 2020). Education also emerged as an important predictor in the prognostic model by Ophey et al. (2022), although exploratory analyses again did not identify a significant direction of effect. Conversely, Kalbe et al. (2020) found lower education to predict greater improvements in executive functioning. Furthermore, one study did not find any significant results for education (Bernini et al., 2021).

#### 3.7.2 Baseline cognitive performance

*Baseline cognitive performance* represented the most frequently investigated predictor and was examined in ten factor finding studies (París et al., 2011; Disbrow et al., 2012; Edwards et al., 2013; Zimmermann et al., 2014; Fellman et al., 2020; Kalbe et al., 2020; Bernini et al., 2021; Ophey et al., 2021; Schmidt et al., 2021; DeGutis et al., 2023). Most studies operationalized baseline cognition as a continuous variable within the same cognitive domain later assessed as an outcome. Disbrow et al. (2012) instead dichotomized participants into “impaired” and “unimpaired” groups according to baseline performance.

Overall, lower baseline executive functioning and working memory were consistently associated with larger post-intervention gains in the same domains (Zimmermann et al., 2014; Kalbe et al., 2020; Ophey et al., 2021; Schmidt et al., 2021). Similarly, PD-MCI showed greater executive functioning improvements than cognitively unimpaired PwPD (París et al., 2011). Disbrow et al. (2012) also reported larger executive-function gains among participants with lower baseline scores. Further, Ophey et al. (2021) found that a lower baseline cognitive performance in working memory predicted greater improvements in the same domain both at post-intervention and follow-up. Associations between baseline cognition and other domains (e.g., memory, language, visuocognition) were less consistent and generally sparse (Kalbe et al., 2016, 2020; Bernini et al., 2021; Schmidt et al., 2021).

In the predictive model (Ophey et al., 2022), baseline verbal working memory and executive functioning emerged as important predictors of intervention responsiveness. Exploratory analyses suggested a positive association between baseline and post-intervention executive functioning performance.

#### 3.7.3 Clinical and genetic factors

*Severity of motor symptoms* was investigated in five factor finding studies. Three studies found no significant associations (Edwards et al., 2013; Bernini et al., 2021; Ophey et al., 2021). In contrast, Kalbe et al. (2020) and Schmidt et al. (2021) reported that lower baseline motor symptom severity predicted greater improvements in motor outcomes after intervention. Additional isolated associations with executive functioning and language were also described.

*LEDD* was investigated in four studies and yielded heterogeneous findings. Higher baseline LEDD predicted greater gains in attention (Edwards et al., 2013) and executive functioning (Kalbe et al., 2020), although these effects diminished or reversed at longer follow-up intervals (Schmidt et al., 2021). Negative associations between LEDD and memory or attention were also observed at follow-up. No significant relationship with working memory was found in Fellman et al. (2020). Nevertheless, LEDD emerged as an important predictor in the prognostic model by Ophey et al. (2022), although exploratory analyses did not clarify the direction of the association.

*Depression* at baseline was assessed as a predictor in five studies. Kalbe et al. (2020) reported that higher depressive symptoms predicted larger reductions in depression post-intervention. Other studies found no significant associations with cognitive or psychological outcomes (Edwards et al., 2013; Bernini et al., 2021; Schmidt et al., 2021). Depression was also included among the relevant predictors identified by Ophey et al. (2022).

*Disease duration* was investigated only in three factor finding studies, yielding heterogeneous results: a positive association with attention (Edwards et al., 2013), a negative association with working memory (Fellman et al., 2020), and no significant relationship with global cognition (Bernini et al., 2021).

When considering *genetic factors*, two studies investigated the predictive role of ApoE4 status and reported conflicting results, with ApoE4 variably associated with both positive and negative cognitive outcomes depending on cognitive domains and follow-up time point (Kalbe et al., 2020; Schmidt et al., 2021).

#### 3.7.4 Intervention-related predictors

The predictive value of *multimodal vs. single-modality interventions* was investigated in three factor finding studies (Reuter et al., 2012; Penko et al., 2019; Rosenfeldt et al., 2019). One study found no significant associations (Penko et al., 2019), whereas the remaining studies suggested that multimodal interventions may produce larger improvements in global cognition, executive functioning, attention, quality of life, and motor symptoms (Reuter et al., 2012; Rosenfeldt et al., 2019).

Three studies compared *analog vs. digital intervention* (De Luca et al., 2019; Bernini et al., 2021; Maggio et al., 2024). Overall, digital interventions were associated with greater improvements in executive functioning and global cognition (Bernini et al., 2021; Maggio et al., 2024), as well as in attention, memory, and language outcomes (De Luca et al., 2019).

The prognostic effect of *unstructured vs. structured interventions* was investigated in three factor finding studies.

Structured interventions were associated with greater improvements in working memory, verbal short-term memory, and attention after CT, and global cognition at the 12-month follow-up (Petrelli et al., 2014, 2015; Bernini et al., 2021). However, unstructured interventions were associated with larger reductions in depressive symptoms immediately after treatment (Petrelli et al., 2014).

#### 3.7.5 Further predictors with highly limited evidence

Several additional predictors were investigated in only one or two studies, limiting the possibility of drawing robust conclusions regarding their prognostic relevance. These included sex (Schmidt et al., 2021; Ophey et al., 2022), fluid intelligence and self-efficacy expectancy (Ophey et al., 2021, 2022), adaptive vs. non-adaptive interventions (Costa et al., 2014; Angelucci et al., 2015), and training dose (Milman et al., 2014; Valdés et al., 2017).

Predictors examined in single studies comprised age at diagnosis (Edwards et al., 2013), in-training baseline working memory intercepts and working memory slopes (Ophey et al., 2022), cognitive reserve (Bernini et al., 2021), side of motor onset (DeGutis et al., 2023), years of drug therapy (Ophey et al., 2022), neuroimaging parameters such as cortical thickness and white matter lesions (Giehl et al., 2024), instrumental activities of daily living (Bernini et al., 2021), self-reported physical activity and quality of life (Schmidt et al., 2021), time of day of intervention administration (Agarwal et al., 2018), analog vs. virtual reality-based interventions (Maggio et al., 2018), and lower vs. higher cognitive load of CT exercises (Fiorenzato et al., 2025).

### 3.8 Quality and risk of bias assessment

The overall risk of bias of each publication, evaluated with the QUIPS checklist (Hayden et al., 2013), presented in Figure 2. Details on individual QUIPS items are provided in Supplementary Table S4. Often, several studies lacked important methodological and reporting information, limiting the interpretability of findings.

**Figure 2:**
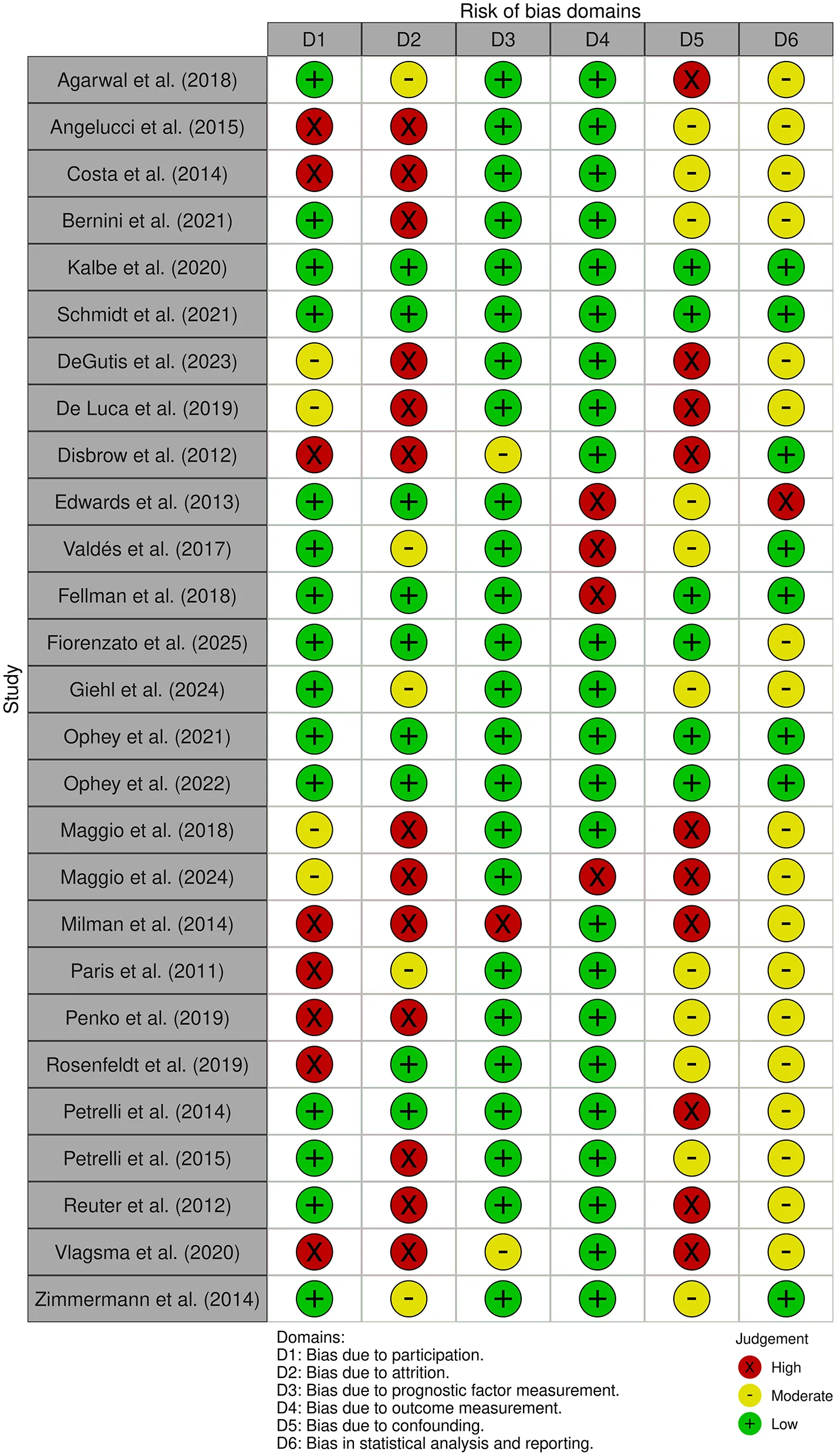
Risk of bias rating. The analyzed domains are based on the Quality in Prognosis Studies (Hayden et al., 2013).

Within the QUIPS domain *Study participation*, common sources of bias included the absence of power analyses (París et al., 2011; Disbrow et al., 2012; Costa et al., 2014; Milman et al., 2014; Angelucci et al., 2015; Agarwal et al., 2018; Maggio et al., 2018, 2024; De Luca et al., 2019; Vlagsma et al., 2020; DeGutis et al., 2023; Fiorenzato et al., 2025) and limited reporting on participant recruitment and selection procedures (París et al., 2011; Costa et al., 2014; Angelucci et al., 2015; Penko et al., 2019; Rosenfeldt et al., 2019; Vlagsma et al., 2020). In this domain, 15 studies were rated as having low risk of bias, four as moderate risk (Maggio et al., 2018,?; De Luca et al., 2019; DeGutis et al., 2023), and eight as high risk (París et al., 2011; Disbrow et al., 2012; Costa et al., 2014; Milman et al., 2014; Angelucci et al., 2015; Penko et al., 2019; Rosenfeldt et al., 2019; Vlagsma et al., 2020).

Regarding the *Study attrition* domain, many studies did not compare baseline characteristics between participants who completed the study and those who dropped out (París et al., 2011; Disbrow et al., 2012; Reuter et al., 2012; Costa et al., 2014; Milman et al., 2014; Angelucci et al., 2015; Agarwal et al., 2018; Maggio et al., 2018, 2024; De Luca et al., 2019; Penko et al., 2019; Kalbe et al., 2020; Vlagsma et al., 2020; Bernini et al., 2021; DeGutis et al., 2023; Fiorenzato et al., 2025). Nine studies were rated as low risk of bias (Edwards et al., 2013; Petrelli et al., 2014; Rosenfeldt et al., 2019; Fellman et al., 2020; Kalbe et al., 2020; Ophey et al., 2021, 2022; Schmidt et al., 2021; Fiorenzato et al., 2025), five as moderate risk (París et al., 2011; Zimmermann et al., 2014; Valdés et al., 2017; Agarwal et al., 2018; Giehl et al., 2024), and the remaining 13 publications as high risk.

The domain *Study confounding* represented one of the most critical methodological weaknesses, as many studies insufficiently accounted for potentially confounding variables (Disbrow et al., 2012; Reuter et al., 2012; Milman et al., 2014; Petrelli et al., 2014; Agarwal et al., 2018; Maggio et al., 2018, 2024; De Luca et al., 2019; Vlagsma et al., 2020; DeGutis et al., 2023). Six publications were rated as low risk of bias (Fellman et al., 2020; Kalbe et al., 2020; Ophey et al., 2021, 2022; Schmidt et al., 2021; Fiorenzato et al., 2025), 10 as high risk (Disbrow et al., 2012; Reuter et al., 2012; Milman et al., 2014; Petrelli et al., 2014; Agarwal et al., 2018; Maggio et al., 2018, 2024; De Luca et al., 2019; Vlagsma et al., 2020; DeGutis et al., 2023), and the remaining studies as moderate risk.

The domains *Prognostic factor measurement and Outcome measurement* were mostly rated as having low risk of bias. Exceptions within *Prognostic factor measurement* included Disbrow et al. (2012) and Vlagsma et al. (2020), which were rated as moderate risk, and Milman et al. (2014), which was rated as high risk. Regarding *Outcome measurement*, four studies were judged to have high risk of bias (Edwards et al., 2013; Valdés et al., 2017; Fellman et al., 2020; Maggio et al., 2024).

The domain *Statistical analyses and reporting* was predominantly rated as moderate risk of bias (18 publications), largely because several studies did not apply statistical methods considered appropriate for prognostic research (Smoleń et al., 2018). In addition, Costa et al. (2014) and Angelucci et al. (2015) did not report that their publications were based on overlapping sample; this information was obtained only after contacting the authors. Eight studies were rated as low risk of bias (Disbrow et al., 2012; Zimmermann et al., 2014; Valdés et al., 2017; Fellman et al., 2020; Kalbe et al., 2020; Ophey et al., 2021, 2022; Schmidt et al., 2021), whereas Edwards et al. (2013) was the only study rated as high risk in this domain.

Taken together, most included publications showed elevated risk of bias in multiple domains, indicating that findings should be interpreted cautiously. Prognostic factor measurement and Outcome measurement were the domains most frequently associated with low risk of bias, whereas Study attrition and Study confounding showed the highest risk across studies. Study participation and Statistical analyses and reporting also emerged as important sources of potential bias.

### 3.9 GRADE assessment results

Overall, the certainty of evidence across outcomes assessed using the GRADE tool (Huguet et al., 2013) ranged from low to very low. Most outcome domains were downgraded because of serious risk of bias and imprecision, reflecting the predominance of exploratory prognostic analyses, heterogeneous statistical approaches, and relatively small sample sizes across studies. Inconsistency was identified for every outcome, due to variability in predictor significance, direction of associations, intervention characteristics, and outcome measures. Indirectness was not considered serious when the outcome was investigated in relation to a representative number of prognostic factors (i.e., executive functions, working memory, memory, attention, global cognition, visuocognition, language, motor functioning, depression). Publication bias was suspected for outcomes supported by one or few small and exploratory studies. For further details, see Supplementary Table S6.

## 4 Discussion

This systematic review aimed to synthetize evidence on the role of individual- and intervention-related factors in predicting responsiveness to cognitive intervention in PwPD. The main results are: (i) the review included 20 trials reported across 27 publications. Twenty-six publications adopted a prognostic factor finding approach, whereas only one developed a prognostic model. (ii) Investigated predictors included baseline cognitive performance, sociodemographic, clinical and genetic factors, and intervention-related features. The most frequently examined predictors were age, education, baseline cognitive performance, severity of motor symptoms, LEDD, depression, and three intervention-related characteristics (i.e., analog vs. computerized, structured vs. unstructured, multimodal vs. single-modality). (iii) across cognitive and non-cognitive outcomes, assessment time points (i.e., post-intervention and follow-up), and methodology including statistical approaches. (iv) For most predictors, evidence was too limited to allow firm conclusions.

Nevertheless, some recurring patterns emerged, most notably that lower baseline cognitive performance was associated with greater cognitive intervention gains. A visual summary of the investigated prognostic factors is presented in Figure 3.

**Figure 3:**
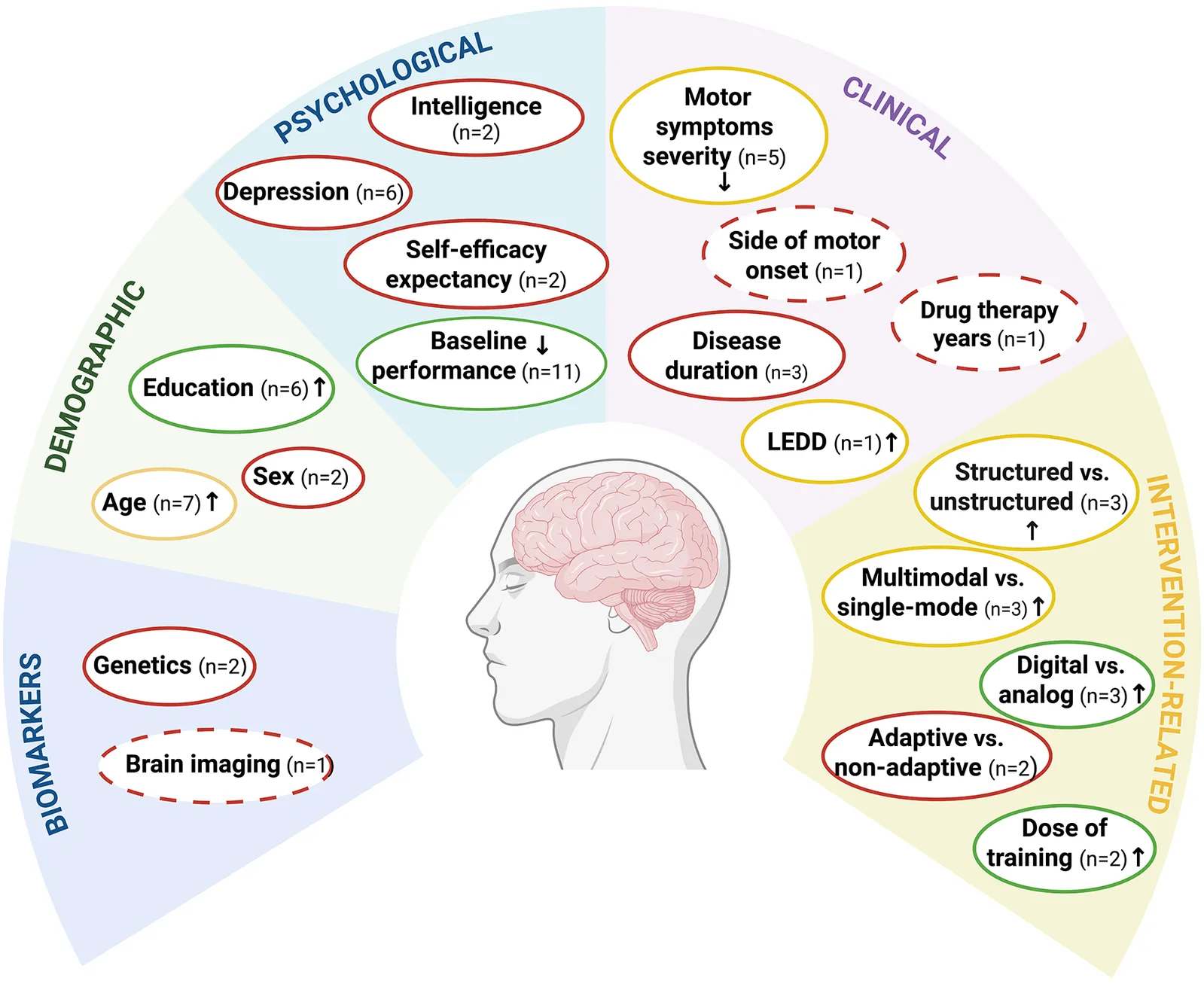
Investigated prognostic factors classified into five clusters. The figure illustrates the examined predictors grouped into five categories (biomarkers, demographic, psychological, clinical, and intervention-related factors). The frequency with which each predictor was investigated is indicated in brackets. Colors reflect the consistency of findings across studies: green indicates consistent associations in the same direction (>80% of findings in the same direction), yellow indicates moderately consistent associations (50%−80%), and red indicates inconsistent findings (<50%). Dotted lines identify predictors investigated in only one publication. Importantly, the illustrated prognostic patterns are based solely on the frequency and direction of reported associations and do not account for methodological quality or risk of bias.

### 4.1 Methodological considerations regarding the included studies

Several methodological considerations have to be considered when interpreting our results. Currently, prognostic research on this topic appears to be scarce and methodologically heterogenous.

As previously noted, prognostic frameworks are often not directly indexed in databases (Riley et al., 2019), and only three included publications explicitly used prediction-related terminology in their titles (Ophey et al., 2021, 2022; DeGutis et al., 2023). In the majority of studies, there was no explicit prognostic research objective.

The statistical approaches applied across studies varied considerably. A frequently used approach consisted of simple correlation analyses between potential prognostic factors and intervention-related outcome changes. However, correlational analyses have been suggested to be suitable mainly for identifying “candidate” prognostic factors and should ideally be followed by more appropriate prognostic approaches, such as latent growth analyses (Smoleń et al., 2018; Kent et al., 2020). Additionally, even more advanced conventional methods, including linear models and multiple regression analyses, may not adequately address prognostic research questions (Smoleń et al., 2018; Ophey et al., 2020). In the present review, only a small number of studies employed statistical approaches specifically suited for prognostic analyses, such as latent growth models and latent difference score models (Valdés et al., 2017; Fellman et al., 2020; Ophey et al., 2021, 2022). Future studies should increasingly adopt these methodologies. Another important limitation concerns the generally small sample sizes of the included studies, which may increase the likelihood of baseline group differences and reduce both internal and external validity (Faber and Fonseca, 2014; Green et al., 2014). Furthermore, power analyses were frequently absent and, when conducted, typically referred only to the primary intervention objective (i.e., CT efficacy) rather than to prognostic analyses. Future studies should therefore define prognostic objectives *a priori* and perform adequately powered analyses specifically targeting prognostic questions. In addition, future prognostic research should more systematically incorporate control groups into statistical analyses.

This would allow to determine whether the relevant predictive factors have an impact in both the group of interest and its control. Notably, the quality and risk of bias assessment conducted in the present review revealed insufficient consideration of potential confounding variables in both study design and statistical analyses. These methodological shortcomings might also have an influence on the results of the prognostic research that should be taken into account. Based on these considerations, a checklist including useful suggestions for future factor finding studies and prognostic models has been defined (Table 4).

**Table 4:** Recommendations for the future development of prognostic factor and prognostic model studies.

1) Set a research question on prognostic factors based on existing literature
2) Design the study following recommendations for prognostic research: –Conduct a power analysis for appropriate sample size considering the prognostic research approach. –Include a control group (passive and/or active) –Adopt adjustment methods (i.e., matching) to control for confounding factors, or account for them in the statistical analysis (e.g., stratification) –Assess various cognitive and non-cognitive (i.e., motor, psychological) outcomes –Operationalize intervention “responsiveness” uniformly (e.g., using change scores)
3) Employ adequate statistical approaches for prognostic analysis (e.g., latent difference score models, growth curve analyses) and perform these on both the experimental and control groups for comparison.
4) Even when exploratory or non-significant, report findings of predictive analyses and sociodemographic and clinical characteristics of both enrolled and dropped-out participants.
5) Be consistent in using the appropriate terminology referring to various types of intervention throughout the publication.

### 4.2 Sociodemographic predictors

Age was among the most frequently investigated prognostic factors. In most studies, however, age did not significantly predict responsiveness to cognitive interventions. Significant findings were reported in Kalbe et al. (2020) and its follow-up by Schmidt et al. (2021) based on their study with CT in individuals with PD-MCI. Besides a few isolated results, these studies suggested an interesting temporal pattern: older participants appeared to benefit most in executive functioning immediately after intervention and at the 6-month follow-up, whereas younger age predicted better executive functioning outcomes at the 12-month follow-up.

The authors interpreted these findings within the theoretical framework proposed by Lövdén et al. (2012). According to the *compensation hypothesis*, individuals with lower baseline performance may show larger intervention gains because they have greater room for improvement, whereas the magnification hypothesis suggests that individuals with better preserved baseline abilities benefit more from intervention. In line with the compensation hypothesis, older PwPD with more pronounced age-related executive dysfunction and neural alterations (Idowu and Szameitat, 2023) may initially derive greater short-term benefits from CT. Conversely, the long-term advantage observed in younger participants may reflect greater preserved neuroplasticity in “younger” older adults (Burke and Barnes, 2006), potentially supporting the maintenance and integration of learned cognitive strategies over time. A similar pattern was observed for memory performance at the 12-month follow-up, possibly mediated by previously acquired executive-function strategies (Higginson et al., 2003; Schmidt et al., 2021).

A negative association between age and training gains was also observed for short-term working memory effects in PwPD without cognitive impairment undergoing digital working memory training (Ophey et al., 2021), suggesting that an intervention at younger age—at least for this type of training which targets fluid intelligence functions—could lead to more intervention benefits. Therefore, findings reported here should be interpreted cautiously as the number of studies investigating age as a prognostic factor in PwPD is too small to draw any sound conclusion. Nevertheless, the current evidence base remains too limited and heterogeneous to allow firm conclusions regarding the prognostic role of age in PwPD.

Higher educational levels appeared more consistently associated with greater cognitive intervention benefits in PwPD. Although Kalbe et al. (2020) reported a negative association between education and post-intervention executive functioning gains, four studies overall suggested that higher education predicted greater responsiveness to intervention (Fellman et al., 2020; Kalbe et al., 2020; Ophey et al., 2021; Schmidt et al., 2021), thereby supporting the magnification hypothesis (Lövdén et al., 2012).

Formal years of education are commonly considered a proxy for cognitive reserve, which is thought to support the recruitment of supplementary or alternative neural pathways to compensate for age-related decline and brain pathology (Stern, 2009). Consistent with this framework, individuals with higher educational attainment may employ more efficient cognitive strategies and enhanced controlled processing during cognitive interventions, resulting in greater intervention gains (Le Carret et al., 2003). Similar associations between education and responsiveness to cognitive interventions have also been reported in healthy older adults (Kim et al., 2021).

Conversely, other evidence reported that a lower education might lead to higher CT benefits (Olazarán et al., 2004; Mondini et al., 2016). However, unlike the studies discussed in the present review, those publications included people affected by non-PD-dementia who had more advanced brain pathology, possibly outweighing the compensatory role of education and cognitive reserve.

### 4.3 Baseline cognitive performance

The most consistent finding across the prognostic factors investigated in the present review was that lower baseline cognitive performance predicted greater cognitive intervention gains. This observation is in line with findings from healthy older adults (López-Higes et al., 2018; Ophey et al., 2020; Roheger et al., 2020a,b). Across studies, lower baseline performance in a cognitive domain was frequently associated with better improvements within the same function after cognitive intervention, both at post-intervention and at follow-up (París et al., 2011; Disbrow et al., 2012; Edwards et al., 2013; Zimmermann et al., 2014; Fellman et al., 2020; Kalbe et al., 2020; Bernini et al., 2021; Ophey et al., 2021; Schmidt et al., 2021; DeGutis et al., 2023). A pattern could be detected for executive functions and working memory, which represented also the most investigated outcomes in the present review (París et al., 2011; Disbrow et al., 2012; Zimmermann et al., 2014; Kalbe et al., 2020; Bernini et al., 2021; Ophey et al., 2021; Schmidt et al., 2021). Similar associations were also observed for memory, visuocognition, and language outcomes (París et al., 2011; Schmidt et al., 2021; Kalbe et al., 2024). From a theoretical perspective, these findings indicate that individuals with high baseline levels have less room for improvement, hence they benefit less from intervention (i.e., compensation hypothesis). However, these findings should be interpreted cautiously. Indeed, the overall certainty of evidence was low to very low according to the GRADE assessment, reflecting methodological heterogeneity, risk of bias, imprecision, and inconsistency across the available literature. As highlighted by Smoleń et al. (2018), negative associations between baseline performance and change scores may partly reflect the statistical phenomenon of regression to the mean, whereby extreme baseline values tend to move closer to the average during repeated measurements (Barnett et al., 2005).

### 4.4 Clinical and genetic factors

When considering the prognostic influence of baseline *severity of motor symptoms* on cognitive intervention efficacy in PwPD, significant findings were sparse and limited to only one trial (Kalbe et al., 2020; Schmidt et al., 2021). In these publications, worse motor symptoms at baseline (i.e., higher UPDRS-III) predicted greater improvements in motor abilities after CT, both in the short- and long-term.

One possible explanation relates to the potential high neuroplasticity of the fronto-striatal circuit (Di Filippo et al., 2009), which is critically involved in both motor and executive functioning in PD. Cognitive interventions targeting these networks may enhance frontal connectivity and compensatory neural mechanisms (Guglietti et al., 2021). Accordingly, PwPD with more pronounced disease-related impairments may have greater potential for improvement following intervention, consistent with the compensation hypothesis (Lövdén et al., 2012).

Similarly, higher baseline levodopa equivalent daily dose (LEDD), often considered an indirect marker of motor symptom burden (Tomlinson et al., 2010), predicted greater improvements in executive functioning (Kalbe et al., 2020) and attention (Edwards et al., 2013) immediately after intervention. Edwards et al. (2013) also reported that longer disease duration predicted greater gains in speed of processing, again suggesting that more clinically vulnerable PwPD may derive greater short-term benefits from CT.

Nevertheless, findings across studies remained inconsistent. For instance, Kalbe et al. (2020) also reported that better baseline motor functioning predicted larger gains in executive functioning and language. In contrast, Fellman et al. (2020) found that longer disease duration negatively predicted working memory improvements. Moreover, ApoE4 carrier status was associated with both greater and smaller CT-related gains depending on the cognitive domain and assessment time point (Kalbe et al., 2020; Schmidt et al., 2021). These heterogeneous findings highlight the preliminary nature of current evidence regarding clinical and genetic predictors of cognitive intervention responsiveness in PwPD.

### 4.5 Intervention-related predictors

Studies comparing *analog vs. digital CT* suggested that digital interventions may lead to greater benefits than analog approaches (De Luca et al., 2019; Bernini et al., 2021; Maggio et al., 2024). This is in line with findings of a recent review (Guglietti et al., 2021), where digital interventions seem to hold an advantage compared to traditional approaches since they are more accessible, especially for PwPD with motor and visuocognitive difficulties, and linked to less dropouts and protocol deviations. Furthermore, by providing real-time feedback, digital interventions could be perceived as more interactive and engaging, and therefore lead to greater improvements (Mohlman et al., 2011). Another possible advantage is the automatic adaptation of task difficulty to the participant's performance level, which may optimize training intensity and individualization.

*Multimodal interventions*, which simultaneously target cognitive and motor abilities, appeared to be more effective than training these functions separately (Penko et al., 2019; Rosenfeldt et al., 2019) or CT alone (Reuter et al., 2012). Engaging in a multimodal intervention seemed to be predictive of improvements in some cognitive (i.e., global cognition, executive functions, and attention) and motor (i.e., arm swing path length, a measure of mobility) outcomes, but it had a non-significant effect on several others (i.e., working memory, verbal fluency, gait velocity, cadence, step length and step width) (Reuter et al., 2012; Penko et al., 2019; Rosenfeldt et al., 2019). However, these results should be interpreted cautiously as the studies were very heterogenous. One trial made a comparison between a multimodal vs. single-modality approach, which targets both the cognitive and motor components but separately (Penko et al., 2019; Rosenfeldt et al., 2019); conversely, Reuter et al. compared a multimodal intervention with a traditional CT (Reuter et al., 2012). Despite this dissimilarity, dual-task training has been generally described as more effective and stimulating compared to a cognitive-only or a single-task intervention, leading to long-lasting involvement and motivation toward the program (Oswald et al., 2006). Furthermore, the integration of cognitive and physical training may be particularly suitable for PwPD given the combined motor and cognitive impairments characteristic of PD (Guglietti et al., 2021).

Only a few publications investigated *structured vs. unstructured* interventions (Petrelli et al., 2014, 2015; Bernini et al., 2021). Overall, structured intervention appears to lead to greater benefits in some cognitive measures (i.e., working memory, verbal short-term memory, attention, global cognition), consistent with findings from healthy older adults (Jaeggi et al., 2008; Roheger et al., 2019). Structured interventions typically consist of standardized exercises targeting one or multiple cognitive domains (Bahar-Fuchs et al., 2013), potentially facilitating the acquisition and consolidation of specific cognitive strategies (Roheger et al., 2019). Interestingly, Petrelli et al. (2014) found that the unstructured approach led to more responsiveness regarding depressive symptoms.

The authors suggested that this effect may have been related to the interactive engaging nature of this specific intervention, which included group discussions and may therefore have positively influenced the affective state of participants.

### 4.6 Strengths and limitations

The present systematic review allowed to identify and fill a current gap in predictive literature, in the context of PwPD, providing the first comprehensive overview of the existing publications. Furthermore, we suggested potentially relevant predictors to further investigate and more fitting statistical choices for future prognostic studies to employ. However, there are also several limitations to take into consideration. First, only three databases for the literature search were employed, so that there is a risk that relevant literature was missed. Second, identifying prognostic studies through systematic literature searches was particularly challenging because prognostic analyses are often not explicitly described in titles, abstracts, or indexing terms. As a result, relevant studies may have been overlooked. Moreover, as discussed in the methodological consideration section, substantial methodological limitations across included studies restrict the possibility of drawing firm conclusions regarding factors that may predict CT responsiveness. In particular, differences in study designs and statistical approaches did not always allow prognostic factors to be clearly distinguished from treatment-effect moderators. Lastly, although not specifically addressed in the present review, PwPD may benefit not only from interventions targeting cognitive abilities but also from treatments addressing psychological symptoms, such as anxiety and depression. Recent studies investigating cognitive-behavioral interventions have shown promising effects on these and related clinical outcomes (e.g., Delshadi et al., 2024; Asadiof et al., 2025). Future prognostic research could therefore investigate factors associated with differential responsiveness to psychological interventions, further informing the personalization of therapeutic approaches for PwPD.

## 5 Conclusions

To sum up, research investigating predictive factors of cognitive intervention responsiveness in PwPD is scarce and inconsistent. The most promising prognostic variable for higher responsiveness seems to be lower baseline cognitive performance. Considering further results showing that more symptom burden in PwPD (e.g., severity of motor symptoms, LEDD) predict CT benefits, a pattern emerges that indicates that more vulnerable PwPD benefit more. Nonetheless, any conclusion must be taken with caution. More studies with high methodological standards regarding prognostic analyses are needed. Investigating which individual or intervention-related characteristics influence response to cognitive interventions in PwPD is essential to implement individualized non-pharmacological interventions, beyond the current “one-treatment-fits-all” approach (Hingorani et al., 2013; Kalbe et al., 2020).

## Data Availability

The original contributions presented in the study are included in the article/Supplementary material, further inquiries can be directed to the corresponding author.

## Supplementary material

Supplementary File S1 Systematic review protocol registered at PROSPERO.

Supplementary Table S2 PRISMA Checklists. Completed PRISMA checklists for abstract and systematic reviews.

Supplementary Table S3 Systematic Search Strategy. Full search strategy for each database (MEDLINE Ovid, CINAHL, Web of Science).

Supplementary Table S4 Risk of Bias Assessment Using the Quality in Prognosis Studies (QUIPS) checklist.

Supplementary Table S5 Intervention characteristics. Detailed characteristics of interventions (i.e., type of intervention, setting, trained cognitive domains, etc.) proposed in each included publication, and time of measurement.

Supplementary Table S6 GRADE assessment (Huguet et al., 2013).
