# Supplementary material for: Prognostic factors for cognitive intervention responsiveness in Parkinson's disease: a systematic review revealing inconsistent methodology and evidence
Source: Front Cognit. 2026 Oct 21;5:1959713. doi: 10.3389/fcogn.2026.1959713 (PMC13730948; doi:10.3389/fcogn.2026.1959713)
Supplement: Supplementary file 1 [file Data_Sheet_1.pdf]

NIHR | National Institute for Health and Care Research

PROSPERO
International prospective register of systematic reviews

Prognostic factors and models for cognitive intervention responsiveness in people with Parkinson's disease: a systematic review

Martina Berri, Angela Colucci, Elke Kalbe, Anja Ophey, Eleonora Fiorenzato, Ann-Kristin Folkerts

Citation
Martina Berri, Angela Colucci, Elke Kalbe, Anja Ophey, Eleonora Fiorenzato, Ann-Kristin Folkerts. Prognostic factors and models for cognitive intervention responsiveness in people with Parkinson's disease: a systematic review . PROSPERO 2025 CRD420251079163. Available from https://www.crd.york.ac.uk/PROSPERO/view/CRD420251079163.

REVIEW TITLE AND BASIC DETAILS

Review title

Prognostic factors and models for cognitive intervention responsiveness in people with Parkinson's disease: a systematic review

Condition or domain being studied

Parkinson's Disease; Cognitive training; Cognitive function

The cognitive spectrum of Parkinson's disease (PD) ranges from cognitively unimpaired performance (PD-NC) to subjective cognitive decline (PD-SCD), mild cognitive impairment (PD-MCI) and PD dementia (PDD) (Aarsland et al., 2021). PD-MCI affects around 40% of people with PD (PwPD, Baiano et al., 2020), and in one third (19%-36%) of the cases, PD-MCI is already present at diagnosis (Yarnall et al., 2014). PDD has a point prevalence of 24% to 31% (Aarsland et al., 2005), and the risk of PDD increases as disease progresses (i.e., from 9% to 27% after 10 years to 74% after 20 years; (Gallagher et al., 2024). SCD is a novel concept in the field of PD research, and findings indicate that it could predict cognitive decline at 2-year follow-up in approximately 30% of PwPD (Erro et al., 2014).

One way to maintain or strengthen cognitive abilities is to undergo cognitive interventions (Mowszowski et al., 2010). Among these, cognitive training (CT), cognitive rehabilitation (CR), and cognitive stimulation can be differentiated (CS, Clare and Woods, 2004).

Rationale for the review

There is growing recognition that individuals respond heterogeneously to cognitive interventions, highlighting the need to understand what underlies this variability. Therefore, identifying predictors that can determine which individuals are likely to benefit from particular cognitive interventions has become essential. This shift in focus supports the transition from one-treatment-fits-all strategies toward more individualized approaches in the context of precision medicine in cognitive care.

However, current research examining prognostic factors in cognitive intervention trials remains limited and inconsistent (Roheger et al., 2021; Ophey et al., 2020). The evidence base is especially sparse when it comes to PD, despite cognitive decline being one of its most impactful non-motor symptoms (Kalbe et al., 2020; Fellman et al., 2020; Ophey et al., 2020). To our knowledge, no systematic review has synthesized the existing prognostic research on cognitive interventions in PD. Consequently, this review aims to fill this gap by illustrating the prognostic factors studied and the predictive models applied to evaluate treatment responsiveness in individuals with PD.

Review objectives

Which factors and models predict responsiveness to cognitive interventions in terms of cognitive outcomes (e.g., executive functions, memory, attention, global cognition, fluid intelligence) and non-cognitive outcomes (e.g., depression, quality of life, motor symptoms) in people with Parkinson's disease?

Keywords

Parkinson's disease; Cognitive training; Cognition; Prognostic factors; Training response

Country

Germany; Italy

ELIGIBILITY CRITERIA

Population

Included

We will include studies with people with a clinical diagnosis of PD of any sex, aged 18 and above, across the overall cognitive spectrum (i.e., cognitively unimpaired as well as people with SCD, MCI, and PDD), although it is not necessary that the cognitive status is made explicit. If patient groups with different underlying diseases will be included within a trial, only studies reporting separate analyses for people with Parkinson's disease will be considered.

Excluded

Prodromal stages of PD (e.g., isolated REM sleep behavior disorder, iRBD) will not be included.

Intervention(s) or exposure(s)

Included

We will assess all studies investigating the effects of cognitive intervention which also examine prognostic factors and models to predict cognitive intervention responsiveness.

Cognitive training (CT), cognitive rehabilitation (CR), as well as cognitive stimulation (CS) interventions with a minimum of 2 sessions in total that target one or multiple cognitive domains will be considered for inclusion. CT is defined as a training program able to provide theoretical

skills and structured practice on tasks reflecting specific cognitive functions (e.g., processing speed, memory, attention, executive functions) (Clare and Woods, 2004). CR is another suitable approach defined as an individualized approach where the therapeutic goals are developed together with the person and their family (Clare and Woods, 2004). CS refers to cognitively stimulating activities, usually carried out in small groups, aiming at stabilizing or improving cognitive and social functioning, specifically in people with dementia (Clare and Woods, 2004).

The cognitive intervention can include analogue and/or computerized tasks with a clear cognitive rationale, which are administered either home-based or in a clinical setting, in individual- or group settings, supervised or unsupervised. Cognitive interventions can be administered alone or in combination with other interventions (e.g., TMS, physical exercise), when they constitute at least 50% of the training.

Comparator(s) or control(s)

This review does not have any comparators

Study design

Both randomized and nonrandomized study types will be included.

Included

According to the checklist for critical appraisal and data extraction for systematic reviews of prediction modelling studies (CHARMS) we will include (Moons et al., 2014):

- Prognostic factor finding studies (i.e., studies aiming at establishing one or several variables as independent prognostic factor(s) associated with an outcome) and correlations studies

- Prognostic model development studies without external validation in independent data

- Prognostic model development studies with external validation in independent data

- External model validation studies with or without model updating

The study design will not be limited to randomized controlled trials (RCTs). Instead, all experimental study designs (e.g., non-randomized controlled trials, single-arm studies) including a cognitive intervention approach with 2 and more sessions will be considered for inclusion.

The analyses of prognostic factors or model does not have to be an explicit objective of the study.

Context

Data on research to predict response to cognitive interventions in people with Parkinson´s disease in all settings (e.g., outpatients, inpatients, home-based, nursing homes, etc.) are included. We include all types of experimental study designs; thus, controlled trials are not mandatory.

TIMELINE OF THE REVIEW

Date of first submission to PROSPERO

27 June 2025

Review timeline

Start date: 21 May 2025. End date: 31 December 2025.

Date of registration in PROSPERO

AVAILABILITY OF FULL PROTOCOL

Availability of full protocol

A full protocol has been written but is not available because:

All relevant review steps including the rationale and objectives were pre-defined when preparing the PROSPERO registration.

SEARCHING AND SCREENING

Search for unpublished studies

Only published studies will be sought.

Main sources that will be searched

The main databases to be searched are *CINAHL - Cumulative Index to Nursing and Allied Health Literature*, *MEDLINE*.

Other important or specialist databases that will be searched
Web of Science

Search language restrictions

The review will only include studies published in English, German and Italian.

Search date restrictions

There are no search date restrictions.

Other methods of identifying studies

Other studies will be identified by: *reference list checking*.

Link to search strategy

A full search strategy has been uploaded to PROSPERO. The PDF may be accessed through this link
https://www.crd.york.ac.uk/PROSPEROFILES/a1f7a2e37d52841fe87479c745ebd166.pdf.

Selection process

Studies will be screened independently by at least two people (or person/machine combination) with a process to resolve differences.

Other relevant information about searching and screening

Identifying prognostic factors and models is generally more challenging as prognostic studies may be carried out through different study designs (e.g., cohort studies, post hoc analyses of RCTs) (Rayner et al., 2024) and may not directly be indexed. In addition, there's a lack of widespread recognition of standardized taxonomy for prognosis research (Riley et al., 2019). Consequently, search strategies are broad and yield a high number of both relevant and irrelevant records, hence reviewers should employ additional searching modalities, such as citation searching, handsearching, or web searching (Rayner et al., 2024).

DATA COLLECTION PROCESS

Data extraction from published articles and reports

Data will be extracted independently by at least two people (or person/machine combination) with a process to resolve differences.

Authors will be asked to provide any required data not available in published reports.

Study risk of bias or quality assessment

Risk of bias will be assessed using:

QUIPS (Hayden et al., 2013)

Data will be assessed independently by at least two people (or person/machine combination) with a process to resolve differences.

Additional information will **not** be sought from study investigators if required information is unclear or unavailable in the study publications/reports.

Reporting bias assessment

Risk of bias due to missing results will not be assessed

Certainty assessment

Certainty of findings will not be assessed

OUTCOMES TO BE ANALYSED

Main outcomes

Short- and long-term response in terms of cognitive outcomes.

Measures of effect

Outcomes need to be measured with established and standardized (neuro-)psychological tests including post-test and follow-up measurements (compared to pre-tests scores).

Additional outcomes

All other outomes, e.g., clinical (including motor symptoms, depression) and quality of life.

Measures of effect

Outcomes need to be measured with established and standardized assessments including post-test and follow-up measurements (compared to pre-tests scores).

PLANNED DATA SYNTHESIS

Strategy for data synthesis

Our aim is to identify, describe, and compare prognostic factors of cognitive intervention responsiveness in people with Parkinson's disease, exploring potential patterns across predictors and outcomes. This will be done by providing a narrative synthesis of the data extracted from the studies. Due to the high heterogeneity of the defined outcomes and the analyzed predictors, no statistical analyses on the extracted data will be conducted.

CURRENT REVIEW STAGE

Stage of the review at this submission

Review stage	Started	Completed
Pilot work	✓	✓
Formal searching/study identification	✓	✓
Screening search results against inclusion criteria	✓	
Data extraction or receipt of IPD		
Risk of bias/quality assessment		
Data synthesis		

Review status
The review is currently planned or ongoing.

Publication of review results
Results of the review will be published in English.

REVIEW AFFILIATION, FUNDING AND PEER REVIEW

Review team members
Mrs Martina Berri. Department of General Psychology, University of Padova, Padova. Italy.

No conflict of interest declared.

Mrs Angela Colucci. Department of General Psychology, University of Padova, Padova. Italy.

No conflict of interest declared.

Professor Elke Kalbe. Medical Psychology | Neuropsychology and Gender Studies & Center for Neuropsychological Diagnostics and Intervention (CeNDI), Faculty of Medicine and University Hospital Cologne, University of Cologne. Germany.

Conflict of interest
EK is author of the cognitive intervention program series NEUROvitalis, but receives no corresponding honoraria.

Dr Anja Ophey. Medical Psychology | Neuropsychology and Gender Studies, Faculty of Medicine and University Hospital Cologne, University of Cologne, Cologne. Germany.

No conflict of interest declared.

Assistant Professor Eleonora Fiorenzato. Parkinson's Disease & Movement Disorders Unit | Department of Neuroscience, University of Padova, Padova. Italy.

No conflict of interest declared.

Dr Ann-Kristin Folkerts (review guarantor and contact) Medical Psychology | Neuropsychology and Gender Studies, Faculty of Medicine and University Hospital Cologne, University of Cologne. Germany.

Conflict of interest
AKF is author of the cognitive intervention program series NEUROvitalis, but receives no corresponding honoraria.

Named contact

Dr Ann-Kristin Folkerts (ann-kristin.folkerts@uk-koeln.de). Medical Psychology | Neuropsychology and Gender Studies, Faculty of Medicine and University Hospital Cologne, University of Cologne. Germany.

Review affiliation

Medical Psychology | Neuropsychology and Gender Studies, Faculty of Medicine and University Hospital Cologne, University of Cologne, Cologne, Germany

Funding source

Review has no specific/external funding but is supported by guarantor/review team (non-commercial) institutions.

Peer review

There has been no peer review of this planned review.

ADDITIONAL INFORMATION

Review conflict of interest

Declared individual interests are recorded under team member details. Two review team members have declared a potential conflict of interest. No additional interests are recorded for this review.

Medical Subject Headings

Cognition; Cognitive Dysfunction; Cognitive Training; Dementia; Depression; Executive Function; Follow-Up Studies; Humans; Parkinson Disease; Processing Speed; Prognosis; Quality of Life; Attention

SIMILAR REVIEWS

Check for similar records already in PROSPERO

PROSPERO identified a number of existing PROSPERO records that were similar to this one (last check made on 23 June 2025). These are shown below along with the reasons given by that the review team for the reviews being different and/or proceeding.

- Prognostic factors and models for working memory training success in healthy older adults: a systematic review and meta-analysis [published 22 October 2019] [CRD42019142750]. The review was judged **not to be similar**
- Prognostic factors and models for benefits of multidomain cognitive training in healthy older adults: a systematic review and meta-analysis [published 28 April 2020] [CRD42020147531]. The review was judged **not to be similar**
- Prognostic factors for memory training success in healthy older adults: a systematic review and meta-analysis [published 16 May 2019] [CRD42019127479]. The review was judged **not to be similar**
- Cognitive training interventions for dementia and mild cognitive impairment in Parkinson's Disease [Cochrane Protocol] [published 22 March 2016] [CRD42016036821]. The review was judged **not to be similar**
- Multicomponent intervention in Mild Cognitive Impairment: A Systematic Review and Meta-analysis. [published 21 February 2021] [CRD42021232373]. The review was judged **not to**

be similar

- Prognostic models for memory training success in healthy older adults: a systematic review and meta-analysis [published 14 August 2018] [CRD42018105803]. The review was judged **not to be similar**
- Cognitive interventions in children with brain tumours and childhood brain cancer survivors– a systematic review and meta-analysis [published 29 June 2021] [CRD42021239839]. The review was judged **not to be similar**
- Predictors of working memory training responsiveness in healthy older adults: an individual participant data meta-analysis [published 4 November 2021] [CRD42021282419]. The review was judged **not to be similar**
- Effects of technology-based cognitive and exercise interventions on cognitive function in persons with mild cognitive impairment: A systematic review, network meta-analysis, and meta regression [published 5 December 2023] [CRD42023486359]. The review was judged **not to be similar**
- Combined physical and cognitive training in older adults: a systematic review and meta-analysis [published 28 April 2020] [CRD42020143509]. The review was judged **not to be similar**
- The Effects of Cognitive-Motor Dual-Task Interventions and Technology on Cognitive Outcomes for Dementia and MCI: A Systematic Review of Systematic Reviews [published 8 April 2022] [CRD42022319845]. The review was judged **not to be similar**
- Cognitive interventions to enhance cognition and quality of life in adult patients with primary brain tumours – a systematic review and meta-analysis [published 25 March 2021] [CRD42021239836]. The review was judged **not to be similar**
- Cognitive training in patients with Parkinson's disease: a systematic review and meta-analysis [published 29 July 2014] [CRD42014012936]. The review was judged **not to be similar**
- Effects of cognitive and combined physical-cognitive training interventions on cognitive, physical, and work performance outcomes in physically active workers: a systematic review and meta-analysis [published 17 June 2025] [CRD420251074662]. The review was judged **not to be similar**
- The Efficacy of Cognitive Intervention programs in amnesic Mild Cognitive Impariment: A Systematic Review [published 5 July 2020] [CRD42020189135]. The review was judged **not to be similar**
- A systematic review and meta-analysis on the effects of multisession tDCS combined with cognitive or motor training [published 13 January 2022] [CRD42022297972]. The review was judged **not to be similar**
- A systematic review and meta-analysis of transfer effects from working memory, video game and meditation-based modalities of cognitive training [published 15 January 2018] [CRD42018084489]. The review was judged **not to be similar**
- Cognitive Training Effectiveness on Memory, Executive Functioning, and Processing Speed in Individuals with Substance Use Disorders: A Systematic Literature Review [published 5 July 2020] [CRD42020161039]. The review was judged **not to be similar**
- Cognitive training for post-acute traumatic brain injury: a systematic review and meta-analysis [published 27 August 2014] [CRD42014013274]. The review was judged **not to be similar**

- Cognitive interventions for older adults: a systematic review and meta-analysis of randomized controlled trials [published 20 May 2016] [CRD42016038386]. The review was judged **not to be similar**

PROSPERO version history

- Version 1.0, published 01 Jul 2025

Disclaimer

The content of this record displays the information provided by the review team. PROSPERO does not peer review registration records or endorse their content.

PROSPERO accepts and posts the information provided in good faith; responsibility for record content rests with the review team. The guarantor for this record has affirmed that the information provided is truthful and that they understand that deliberate provision of inaccurate information may be construed as scientific misconduct.

PROSPERO does not accept any liability for the content provided in this record or for its use. Readers use the information provided in this record at their own risk.

Any enquiries about the record should be referred to the named review contact
