# Supplementary material for: Prognostic factors for cognitive intervention responsiveness in Parkinson's disease: a systematic review revealing inconsistent methodology and evidence
Source: Front Cognit. 2026 Oct 21;5:1959713. doi: 10.3389/fcogn.2026.1959713 (PMC13730948; doi:10.3389/fcogn.2026.1959713)
Supplement: Supplementary file 3 [file Data_Sheet_3.pdf]

Supplementary Table S3. Systematic Search Strategy. Full search strategy for each database (MEDLINE Ovid, CINAHL, Web of Science).

2.1 CINHAL

Access: 21.05.2025 Results: 3069

#	Query	Limiters/Expanders	Last Run Via	Results
S8	S3 AND S7	Expanders - Apply equivalent subjects Search modes - Proximity	Interface - EBSCOhost Research Databases Search Screen - Advanced Search Database - CINAHL	3,069
S7	S4 OR S5 OR S6	Expanders - Apply equivalent subjects Search modes - Proximity	Interface - EBSCOhost Research Databases Search Screen - Advanced Search Database - CINAHL	64,411
S6	TX (((brain* N2 train*) OR (brain* N2 intervent*) OR (brain* N2 exercis*) OR (brain* N2 rehabilitat*) OR (brain* N2 stimulat*) OR (brain* N2 retrain*)))	Expanders - Apply equivalent subjects Search modes - Proximity	Interface - EBSCOhost Research Databases Search Screen - Advanced Search Database - CINAHL	16,345
S5	TX (((cognit* N2 train*) OR (cognit* N2 intervent*) OR (cognit* N2 therap*) OR (cognit* N2 exercis*) OR (cognit* N2 rehabilitat*) OR (cognit* N2 stimulat*) OR (cognitive N2 support) OR (cognit* N2 retrain*)))	Expanders - Apply equivalent subjects Search modes - Proximity	Interface - EBSCOhost Research Databases Search Screen - Advanced Search Database - CINAHL	46,162
S4	TX (((memory* N2 train*) OR (memory* N2 intervent*) OR (memory* N2 exercis*) OR (memory* N2 rehabilitat*) OR (memory* N2 stimulat*) OR (memory* N2 therap*) OR (memory* N2 retrain*) OR (memory* N2 support) OR (memory* N2 aid*) OR (memory* N2 group*) OR (memory* N2 strateg*) OR (memory* N2 management)))	Expanders - Apply equivalent subjects Search modes - Proximity	Interface - EBSCOhost Research Databases Search Screen - Advanced Search Database - CINAHL	4,596

#	Query	Limiters/Expanders	Last Run Via	Results
S3	S1 OR S2	Expanders - Apply equivalent subjects Search modes - Proximity	Interface - EBSCOhost Research Databases Search Screen - Advanced Search Database - CINAHL	44,287
S2	TX parkinson*	Expanders - Apply equivalent subjects Search modes - Proximity	Interface - EBSCOhost Research Databases Search Screen - Advanced Search Database - CINAHL	44,287
S1	(MH "Parkinson Disease")	Expanders - Apply equivalent subjects Search modes - Proximity	Interface - EBSCOhost Research Databases Search Screen - Advanced Search Database - CINAHL	25,475

2.2 MEDLINE Ovid

Search string modified from Orgeta et al. (2020).

Access: 15.05.2025

Results: 9063

1 parkinson*.ti,ab

2 exp Parkinson's Disease/

3 exp Parkinsonism/

4 or/1-3

5 *Cognitive Therapy/

6 (cognit* adj2 therapy).ti,ab.

7 (cognit* adj2 intervention).ti,ab.

8 (cognit* adj2 training).ti,ab.

9 (cognit* adj2 rehabilitation).ti,ab.

10 (cognit* adj2 stimulation).ti,ab.

11 (cognit* adj2 retrain*).ti,ab.

12 "cognitive support".ti,ab.

13 (memory adj2 therap*).ti,ab.

14 (memory adj2 intervention).ti,ab.

15 (memory adj2 training).ti,ab.

16 (memory adj2 rehabilitation).ti,ab.

17 (memory adj2 stimulation).ti,ab.

18 (memory adj2 retrain*).ti,ab.

19 "memory support".ti,ab.

20 "memory aid*".ti,ab.

21 "memory group*".ti,ab.

22 "memory strateg*".ti,ab.

23 "memory management".ti,ab.

24 (brain adj2 therapy).ti,ab.

25 (brain* adj2 intervention).ti,ab.

26 (brain* adj2 training).ti,ab.

27 (brain* adj2 rehabilitation).ti,ab.

28 (brain adj2 stimulation).ti,ab.

29 (brain adj2 retrain*).ti,ab.

30 or/5-29

31 4 and 30

2.3 Web of Science

Search string modified from Orgeta et al. (2020).

Access: 15.05.2025

Results: 10043

TOPIC: (parkinson* OR "PD")

AND

TOPIC: ("cognit* train*" OR "cognit* intervent*" OR "cognit* exercis*" OR "cognit* rehabilitat*" OR "cognit* stimulat*" OR "cognit* therap*" OR "cognit* retrain*" OR "cognitive support" OR "memory train*" OR "memory intervent*"OR "memory exercis*" OR "memory rehabilitat*" OR "memory stimulat*" OR "memory therap*" OR "memory retrain*" OR "memory support" OR "memory aid*" OR "memory group*" OR "memory strateg*" OR "memory management" OR "brain train*" OR "brain intervent*" OR "brain exercis*" OR "brain rehabilitat*" OR "brain stimulat*" OR "brain retrain*")
