# Supplementary material for: Prognostic factors for cognitive intervention responsiveness in Parkinson's disease: a systematic review revealing inconsistent methodology and evidence
Source: Front Cognit. 2026 Oct 21;5:1959713. doi: 10.3389/fcogn.2026.1959713 (PMC13730948; doi:10.3389/fcogn.2026.1959713)
Supplement: Supplementary file 4 [file Data_Sheet_4.pdf]

Supplementary Table S4. Risk of Bias Assessment Using the Quality in Prognosis Studies (QUIPS) checklist.

Domains and subdomains assessed with the QUIPS tool	Issues to consider according to the QUIPS tool and judgment reasons of the review authors
**Study participation**	The domain was rated with high risk, if no inclusion or exclusion criteria were stated, or if more than two subdomains were rated as "high risk". It was rated as "moderate risk", if two domains were rated with a "high risk".
Source of target population	The source population or population of interest is adequately described.
Method used to identify population	The sampling frame and recruitment are adequately described, including methods to identify the sample sufficient to limit potential bias.
Recruitment period	The period of recruitment was described.
Place of recruitment	Place of recruitment (setting and geographic location) are adequately described.
Inclusion and exclusion criteria	Inclusion and exclusion criteria are adequately described (e.g., including explicit diagnostic criteria or "zero time" description).
Adequate study participation	There is adequate participation in the study by eligible individuals.
Baseline characteristics	The baseline study sample (i.e., individuals entering the study) is adequately described for at least the variables age, sex, and education.
**Study Attrition**	The domain was rated with high risk, if more than two subdomains were rated as "high risk". It was rated as "moderate risk", if two domains were rated with a "high risk" or if either "Reasons for lost to follow-up" or "Outcome and prognostic factor information" was rated with a "high risk".
Proportion of baseline sample available for analysis	Response rate (i.e., proportion of study sample completing the study and providing outcome data) is adequate.
Attempts to collect information on participants who dropped out	Attempts to collect information on participants who dropped out of the study are described.
Reasons and potential impact of subjects lost to follow-up	Reasons for loss to follow-up are provided.
Outcome and prognostic factor information on those lost to follow-up	Participants lost to follow-up are adequately described, for at least age, sex, and education. There are no important differences between key characteristics (age, sex, education) and outcomes in participants who completed the study and those who did not.
**Prognostic Factor Measurement**	The domain was rated with high risk, if more than two subdomains were rated as "high risk" or if the subdomain "definition of prognostic factor" was

	rated as “high risk”. It was rated as “moderate risk”, if two domains were rated with a “high risk”.
Definition of the PF	A clear definition or description of 'PF' is provided.
Valid and Reliable Measurement of PF	The method of PF measurement is adequately valid and reliable to limit misclassification bias. Continuous variables are reported or appropriate cut-points (i.e., not data-dependent) are used.
Method and Setting of PF Measurement	The method and setting of measurement of PF is the same for all study participants.
Proportion of data on PF available for analysis	An adequate proportion of the study sample has complete data for the PF variable.
Method used for missing data	Appropriate methods of imputation are used for missing 'PF' data.
**Outcome Measurement**	The domain was rated with “high risk”, if one subdomain was rated as “high risk”.
Definition of the Outcome	A clear definition of outcome is provided, including duration of follow-up and level and extent of the outcome construct.
Valid and Reliable Measurement of Outcome	The method of outcome measurement used is adequately valid and reliable to limit misclassification bias.
Method and Setting of Outcome Measurement	The method and setting of outcome measurement is the same for all study participants.
**Study Confounding**	The domain was rated with “high risk”, if two or more subdomains were rated as “high risk”. The domain was rated with “medium risk” if one domain was rated with “high risk”.
Important Confounders Measured	Important confounders, including treatments, are measured.
Definition of the confounding factor	Clear definitions of the important confounders measured are provided.
Valid and Reliable Measurement of Confounders	Measurement of all important confounders is adequately valid and reliable (e.g., may include relevant outside sources of information on measurement properties, also characteristics, such as blind measurement and limited reliance on recall).
Method and Setting of Confounding Measurement	The method and setting of confounding measurement are the same for all study participants.
Method used for missing data	Appropriate methods are used if imputation is used for missing confounder data.
Appropriate Accounting for Confounding	Important potential confounders are accounted for in the study design or in the analysis.
**Statistical Analysis and reporting**	The domain was rated with “high risk” if the subdomain “Presentation of analytical strategy” was

	rated as “high risk” or if more than one of the other subdomains was rated with “high risk”. The domain was rated with “medium risk” if one subdomain (except the first) was rated with “high risk”.
Presentation of analytical strategy	There is sufficient presentation of data to assess the adequacy of the analysis.
Model development strategy	The strategy for model building (i.e., inclusion of variables in the statistical model) is appropriate and is based on a conceptual framework or model. The selected statistical model is adequate for the design of the study (e.g. regression model, mixed models).
Reporting of results	There is no selective reporting of results.

Nach Hayden, J. A., van der Windt, D. A., Cartwright, J. L., Côté, P., & Bombardier, C. (2013). Assessing bias in studies of prognostic factors. Annals of Internal Medicine, 158(4), 280-286. Adapted from: Ophey, A., Roheger, M., Folkerts, A.-K., Skoetz, N., & Kalbe, E. (2020). A Systematic Review on Predictors of Working Memory Training Responsiveness in Healthy Older Adults: Methodological Challenges and Future Directions. *Frontiers in Aging Neuroscience*, *12*. https://doi.org/10.3389/fnagi.2020.575804
