# Supplementary material for: Prognostic factors for cognitive intervention responsiveness in Parkinson's disease: a systematic review revealing inconsistent methodology and evidence
Source: Front Cognit. 2026 Oct 21;5:1959713. doi: 10.3389/fcogn.2026.1959713 (PMC13730948; doi:10.3389/fcogn.2026.1959713)
Supplement: Supplementary file 5 [file Data_Sheet_5.pdf]

Supplementary Table S5. Intervention characteristics. Detailed characteristics of interventions (i.e., type of intervention, setting, trained cognitive domains, etc.) proposed in each included publication, and time of measurement.

**Datasets**		**Study**	**Intervention**					**Time points of measurement**
		**Author (year)**	**Type of intervention**	**Total time (in minutes) & frequency of intervention**	**Setting of intervention**	**Trained cognitive domains**	**Description of intervention**	
	1	Agarwal et al. (2018)	CT+ tDCS	200 (ten sessions of 20 min five times per week over two weeks)	Digital individual tasks	Multiple	EG: computerized games specially selected for attention, processing speed and working memory, combined with remotely supervised-tDCS over bilateral DLPFC	Post-intervention
	2a	Angelucci et al. (2015)	CT	540 (twelve sessions of 45 min three times per week over 4 weeks)	Analog individual tasks	Single (EF)	EG: adaptive exercises grouped in 4 modules, each requiring 3 sessions. Subjects were asked to alternately select between stimuli with different visual and spatial features or according to their belonging to different semantic categories *vs* CG: non-adaptive simple cognitive exercises for sustained attention and language abilities (dictation exercises and reordering of sentences sequences) and respiratory exercises (half of the session)	Post-intervention
	2b	Costa et al. (2014)	CT	540 (twelve sessions of 45 min three times per week over 4 weeks)	Analog individual tasks	Single (EF)	EG: adaptive exercises grouped in 4 modules, each requiring 3 sessions. Subjects were asked to alternately select between stimuli with different visual and spatial features or according to their belonging to different semantic categories *vs* CG: non-adaptive simple cognitive exercises for sustained attention and language abilities (dictation exercises and reordering of sentences sequences) and respiratory exercises (half of the session)	Post-intervention
	3	Bernini et al. (2021)	CT	540 (twelve sessions of 45 min four times a week over three weeks)	Digital *vs* analog individual tasks	Multiple	EG1: computerized training (CoRE) -11 tasks targeting several cognitive abilities (e.g., logical-executive functions, attention/processing speed, working memory, and episodic memory), where thresholds are defined to allow difficulty levels to be progressively increased *vs* EG2: paper-and pencil versions of the task employed in CoRE *vs* CG*:* unstructured activities (reading magazines, chatting, drawing)	Post-intervention
**ID: DRKS00010186**	4a	Kalbe et al. (2020)	CT	1080 (twelve sessions of 90 min twice a week over six weeks)	Analog individual and group tasks	Multiple	NEUROvitalis (EG): individual tasks, group tasks and group games focusing on attention, memory, executive functions, visuocognition and a psychoeducational part *vs* low-intensity physical activity program (CG): warm-up exercises, exercises focusing on stretching, flexibility, loosening up, or relaxation, psychoeducation.	Post-intervention

Datasets		Study	Intervention					Time points of measurement
	4b	Schmidt et al. (2021)	CT	1080 (twelve sessions of 90 min twice a week over six weeks)	Analog individual and group tasks	Multiple	NEUROvitalis (EG): individual tasks, group tasks and group games focusing on attention, memory, executive functions, visuocognition and a psychoeducational part *vs* low-intensity physical activity program (CG): warm-up exercises, exercises focusing on stretching, flexibility, loosening up, or relaxation, psychoeducation.	Follow-up at 6 (FU-1) and 12 months (FU-2)
	5	DeGutis et al. (2023)	CT	720 (twenty sessions of 36 min five times a week over 4 weeks)	Digital individual tasks	Single (EF)	CT: adaptive cognitive training program (Tonic and Phasic Alertness Training - TAPAT) that targets sustained attention by having participants practice focusing their attention and filtering out distractors	Post-intervention and follow-up at 1 month
	6	De Luca et al. (2019)	CR	1440 (24 sessions of 60 minutes three times per week over eight weeks)	Digital individual tasks	Multiple	EG: adaptive computerized cognitive exercises (i.e. attention, memory, spatial cognition, and verbal and non-verbal executive functions) using ERICA platform *vs* CG: standard cognitive training (SCT), based on a face-to-face interaction between therapist and patient and on paper and pencil activities	Post-intervention
	7	Disbrow et al. (2012)	CR	400 (ten sessions of 40 min five days a week over 12-14 days)	Digital individual tasks	Single (EF)	CR: adaptive conditions - (i) explicit informative visual cue to generate a motor plan (EC condition) - participants were presented with a sequence of numbers consisting of the digits 1, 2, 3 and 4 corresponding to the index, middle, ring and little fingers, and were required to type the sequence corresponding to the visual number cues; (ii) uninformative minimal visual cue, where sequence was internally represented (IR condition) - following successful production of the sequence, a green circle appeared at the center of the screen after an interval with no stimulus on the screen, indicating that subjects must produce the sequence from memory	Post-intervention

**Datasets**		**Study**	**Intervention**					**Time points of measurement**
**ID: NCT0115 5349**	8a	Edwards et al. (2013)	CT	1200 (twenty sessions of 60 min three times per week over 12 weeks)	Digital individual tasks	Single (Speed of processing)	EG: 5 digitalized InSight program with 5 exercises designed to improve information processing speed in realistic visual contexts. Across exercises, the visual stimuli change in a graduated fashion, while within exercises, the stimuli become degraded or less discriminable, and speed of presentation increases as user performance improves *vs* CG: waiting period	Post-intervention
	8b	Valdés et al. (2017)	CT	1200 (twenty sessions of 60 min three times per week over 3 months)	Digital individual tasks	Single (Speed of processing)	EG: 5 digitalized InSight program with 5 exercises designed to improve information processing speed in realistic visual contexts. Across exercises, the visual stimuli change in a graduated fashion, while within exercises, the stimuli become degraded or less discriminable, and speed of presentation increases as user performance improves *vs* CG: delayed intervention	Follow-up at 3 months
	9	Fellman et al. (2018)	CT	450 (fifteen sessions of 30 min three times per week over 5 weeks)	Digital individual tasks	Single (WM)	EG: adaptive home-based online WM training with three different WM tasks - n-back, Selective Updating of Sentences Training task (SUST), a Forward Simple Span Training task *vs* CG: adaptive home-based free online quiz task (Älypää) of general knowledge questions	Post-intervention
	10	Fiorenzato et al. (2025)	CT	360 (twelve sessions of 30 min three times per week over 4 weeks)	Virtual reality individual tasks	Single (EF)	EG: adaptive cognitive tasks (Planning, Updating, and Shifting) performed in an iVR environment designed to simulate a real-life supermarket vs CG: adaptive cognitive tasks (placebo-Planning, placebo-Updating, and placebo-Shifting) with lower cognitive load performed in an iVR environment designed to simulate a real-life supermarket	Post-intervention and follow-up at 2 months

Datasets		Study	Intervention					Time points of measurement
**ID: DRKS00009379**	11a	Giehl et al. (2024)	CT	750 (twenty-five sessions of 30 min once a day for five days a week over five weeks)	Digital individual tasks	Single (WM)	EG: computerized WMT (NeuroNation) - 5 varying WM tasks per training session. Every session began with a 4-min warm-up task, followed by four 6.5-min training tasks. The WMT was adapted to user performance across training sessions, home-based and individually accessed online vs CG: waiting list	Post-intervention and follow-up at 3 months
	11b	Ophey et al. (2021)	CT	750 (twenty-five sessions of 30 min once a day for five days a week over five weeks)	Digital individual tasks	Single (WM)	EG: computerized WMT (NeuroNation) - 5 varying WM tasks per training session. Every session began with a 4-min warm-up task, followed by four 6.5-min training tasks. The WMT was adapted to user performance across training sessions, home-based and individually accessed online vs CG: waiting list	Post-intervention and follow-up at 3 months
	11c	Ophey et al. (2022)	CT	750 (twenty-five sessions of 30 min once a day for five days a week over five weeks)	Digital individual tasks	Single (WM)	EG: computerized WMT (NeuroNation) - 5 varying WM tasks per training session. Every session began with a 4-min warm-up task, followed by four 6.5-min training tasks. The WMT was adapted to user performance across training sessions, home-based and individually accessed online vs CG: waiting list	Post-intervention and follow-up at 3 months
	12	Maggio et al. (2018)	CR	1440 (24 sessions of 60 minutes three times per week over 8 weeks)	Virtual reality individual tasks	Multiple	EG: motor and CR via BTS-Nirvana, a semi-immersive therapy system creating virtual scenarios allowing adaptive “interactive training” (especially of executive functions, attention, visuo-spatial skills) for the patient, guided by the therapist *vs* CG: (CTT) face-to-face interaction between therapist and patient and paper-and-pencil activities	Post-intervention

Datasets		Study	Intervention					Time points of measurement
	13	Maggio et al. (2024)	CT	540 (eighteen sessions of 30 min three times per week over six weeks)	Digital individual tasks	Multiple	EG1: two cognitive apps (NeuroNation - Brain Training enhances cognitive performance across multiple cognitive domains; Train Your Brain focuses on concentration, spatial thinking, and reasoning skills) vs EG2: two cognitive apps (Neuronation-Brain Training, plus the app "The Sims™ Mobile" - patient overcomes social challenges with audiovisual feedback) vs CG: worksheets with exercises for cognitive and emotional social components (puzzles and memory tasks)	Post-intervention and follow-up at 3 months
	14	Milman et al. (2014)	CT	1080 (thirty-six sessions of at least 30 min three times per week over 12 weeks)	Digital individual tasks	Multiple	EG: Attengo® software, a computer-based cognitive remediation program, was used to improve cognition, focusing on executive function and attention. After an automated, personalized assessment of the subject's cognitive abilities, the program was tuned to each patient's needs.	Post-intervention and follow-up at 1 month
	15	Paris et al. (2011)	CT	540 (twelve sessions of 45 min three times per week over 4 weeks + exercise to do at home once per week)	Analog and digital individual tasks	Multiple	EG: interactive multimedia software (SmartBrain) targeting cognitive domains such as attention/WM, memory, psychomotor speed, EF, and visuospatial abilities) and nonspecific cognitive exercises, together with paper-and-pencil exercises + cognitive homework exercises *vs* CG: speech therapy to make participants aware of their speech and communication difficulties	Post-intervention
**ID: NCT02538029**	16a	Penko et al. (2019)	Multimodal training (CT + motor training)	1080 (twenty-four sessions of 45 minutes three times per week over 8 weeks)	Analog individual training	Multiple	Dual-task group (MMT): simultaneous gait and cognitive training (executive function, attention, memory, and language tasks) *vs* Single-task group (SMT): evenly divided session between gait and cognitive training (executive function, attention, memory, and language tasks)	Post-intervention and follow-up at one month

Datasets		Study	Intervention					Time points of measurement
	16b	Rosenfeldt et al. (2019)	Multimodal training (CT + motor training)	1080 (twenty-four sessions of 45 minutes three times per week over 8 weeks)	Analog individual training	Multiple	Dual-task group (MMT): simultaneous gait and cognitive training (executive function, attention, memory, and language tasks) vs Single-task group (SMT): evenly divided session between gait and cognitive training (executive function, attention, memory, and language tasks)	Post-intervention and follow-up at one month
**LANDSCAPE**	17a	Petrelli et al. (2014)	CT	1080 (twelve sessions of 90 min twice a week over 6 weeks)	Analog individual and group tasks	Multiple	EG1 (NEUROvitalis): individual tasks, group tasks and group games focusing on attention, memory, executive functions and a psychoeducational part vs EG2 (Mentally Fit): individual and group tasks training attention, memory, language, creative thinking and group conversations about topics proposed by trainer or patients	Post-intervention
	17b	Petrelli et al. (2015)	CT	1080 (twelve sessions of 90 min twice a week over 6 weeks)	Analog individual and group tasks	Multiple	EG1 (NEUROvitalis): individual tasks, group tasks and group games focusing on attention, memory, executive functions and a psychoeducational part *vs* EG2 (Mentally Fit): individual and group tasks training attention, memory, language, creative thinking and group conversations about topics proposed by trainer or patients	Follow-up at 12 months
	18	Reuter et al. (2012)	Multimodal training (CT + motor training)	four sessions of 60 min per week - at least 14 sessions in total + 6-months at-home training (not completed by all patients)	Mixed	Multiple	EG1: CR with training of attention, concentration, biographical work, reasoning, memory, working memory, social rules, cognitive information speed, prospective memory, cognitive estimation, problem solving, planning, associations, and coping with disease *vs* EG2: CR + transfer training which required patients to practice competence in tasks of daily routines with the aim to support them in managing daily life (concentration, strategy, orientation, planning, use of mnemonic devices) *vs* EG3: CR + transfer training + motor training with tasks to enhance inhibitory control, working memory, attention, visuospatial abilities, planning, and motor skills such as coordination, strength, speed, perception, and orientation	Post-intervention and follow-up at 6 months

Datasets		Study	Intervention					Time points of measurement
	19	Vlagsma et al. (2020)	CR	Fourteen sessions of 60 min once or twice a week	Analog *vs* digital individual tasks	Single (EF)	EG: individual strategic executive treatment (Strategic Executive Treatment - ReSET) where patients are taught strategies to compensate for impairments in EF in everyday life situations (formulation of intentions and actions explicitly in terms of goals and sub goals, and effectively executing these plans, while monitoring their behaviour) *vs* CG: CT program (Cogniplus) aimed at training aspects of attention and working memory	Post-intervention (T1) follow-up at 3-5 months
	20	Zimmermann et al. (2014)	CT	480 (twelve sessions of 40 min three times per week over 4 weeks)	Digital individual tasks	Multiple	EG: adaptive training program (CogniPlus) with modules for specific cognitive abilities - focused attention; working memory; planning and action skills; response inhibition *vs* CG: adaptive Nintendo Wii training performed by playing 4 sports games from Wii Sports Resort- Table Tennis, Swordplay, Archery, and Air Sports	Post-intervention

Notes: §, group of participants discussed in the present systematic review; ACE, Addenbrooke's Cognitive Examination; ADL, Activities of Daily Living; BDI-II, Beck Depression Inventory; CG, control group; CR, cognitive rehabilitation; CT, cognitive training; DemTect, Demenz-Test; dlPFC, dorso-lateral prefrontal cortex; EG, experimental group; GDS, Geriatric Depression Scale; HADS-D, Hospital Anxiety and Depression Scale; H&Y, Hoehn & Yahr; I-PD (impaired-PD); iVR, immersive Virtual Reality; LEDD, Levodopa Daily Dose; LPD, left side of motor onset; MMP, Mini-Mental Parkinson's Test; MMSE, Mini-Mental State Examination; MMT, multimodal training group; MoCA, Montreal Cognitive Assessment; n, number of participants; n.a., not applicable; PD, Parkinson's Disease; PDD (PD dementia); PD-MCI (PD-Mild Cognitive Impairment); PD-NC (PD-Normal Cognition); PANDA, Parkinson Neuropsychometric Dementia Assessment; RCT, randomized controlled trial; RPD, right side of motor onset; SCOPA-COG, SCales for Outcomes in PArkinson's disease-COGnition; SMT, single-modality training group; tDCS, transcranial Direct Current Stimulation;TELE, Telephone Screening Protol;TICS-M, Telephone Interview for Cognitive Status-Modified; U-PD (unimpaired-PD); UPDRS, Unified Parkinson's Disease Rating Scale; WAIS-II, Wechsler Adult Intelligence Scale II; WM, working memory; WMT, working memory training
