# Supplementary material for: Prognostic factors for cognitive intervention responsiveness in Parkinson's disease: a systematic review revealing inconsistent methodology and evidence
Source: Front Cognit. 2026 Oct 21;5:1959713. doi: 10.3389/fcogn.2026.1959713 (PMC13730948; doi:10.3389/fcogn.2026.1959713)
Supplement: Supplementary file 6 [file Data_Sheet_6.pdf]

Supplementary Table S6. GRADE Assessment

N of studies	Certainty assessment						Effect			Certainty	Importance
	**Study design**	**Risk of bias**	**Inconsistency**	**Indirectness**	**Imprecision**	**Other considerations**	**N of events**	**N of individuals**	**Rate (95% CI)**		
Executive functions											
17	randomised trials	serious[a]	serious[b]	not serious	serious[c]	none	-	694		⊕◯◯◯ Very low[a,b,c]	
Working Memory											
11	randomised trials	serious[d]	serious[b]	not serious	serious[c]	none	-	330		⊕◯◯◯ Very low[b,c,d]	
Memory											
10	randomised trials	serious[a]	serious[b]	not serious	serious[c]	none	-	334		⊕◯◯◯ Very low[a,b,c]	
Prospective memory											
2	randomised trials	serious[e]	serious[e]	very serious[f]	serious[g]	publication bias strongly suspected[h]	-	47		⊕◯◯◯ Very low[e,f,g,h]	
Attention											
14	randomised trials	serious[a]	serious[b]	not serious	serious[c]	none	-	503		⊕◯◯◯ Very low[a,b,c]	
Global cognition											
9	randomised trials	serious[a]	serious[b]	not serious	serious[c]	none	-	484		⊕◯◯◯ Very low[a,b,c]	
Visuocognition											
6	randomised trials	serious[a]	serious[b]	not serious	serious[c]	publication bias strongly suspected[i]	-	245		⊕◯◯◯ Very low[a,b,c,i]	
Language											
3	randomised trials	serious[a]	serious[b]	not serious	serious[c]	publication bias strongly suspected[h]	-	121		⊕◯◯◯ Very low[a,b,c,h]	
Motor functioning											
8	randomised trials	very serious[j]	serious[b]	not serious	serious[c]	publication bias strongly suspected[h]	-	448		⊕◯◯◯ Very low[b,c,h,j]	

N of studies	Certainty assessment						Effect			Certainty	Importance
	Study design	Risk of bias	Inconsistency	Indirectness	Imprecision	Other considerations	N of events	N of individuals	Rate (95% CI)		
ADL / IADL (Activities of Daily Living / Instrumental Activities of Daily Living)											
4	randomised trials	serious[a]	serious[b]	serious[k]	serious[c]	publication bias strongly suspected[h]	-	124		⊕◯◯◯ Very low[a,b,c,h,k]	
Self-reported Physical Activity											
2	randomised trials	serious[a]	serious[b]	not serious	very serious[l]	publication bias strongly suspected[h]	-	61		⊕◯◯◯ Very low[a,b,h,l]	
Quality of Life											
5	randomised trials	serious[a]	serious[b]	serious[k]	serious[c]	publication bias strongly suspected[h]	-	380		⊕◯◯◯ Very low[a,b,c,h,k]	
Depression											
5	randomised trials	serious[a]	serious[b]	not serious	serious[c]	publication bias strongly suspected[h]	-	204		⊕◯◯◯ Very low[a,b,c,h]	
Self-experienced attention deficits											
2	randomised trials	serious[e]	serious[b]	serious[k]	very serious[l]	publication bias strongly suspected[h]	-	76		⊕◯◯◯ Very low[b,e,h,k,l]	
Brain-Derived Neurotrophic Factor											
1	randomised trials	very serious[m]	serious[g]	very serious[f]	very serious[g]	publication bias strongly suspected[h]	-	15		⊕◯◯◯ Very low[f,g,h,m]	
Phonemic fluency											
4	randomised trials	serious[m]	serious[n]	very serious[f]	serious[o]	publication bias strongly suspected[h]	-	137		⊕◯◯◯ Very low[f,h,m,n,o]	
Semantic fluency											
1	randomised trials	serious[e]	serious[n]	very serious[f]	very serious[o]	publication bias strongly suspected[h]	-	17		⊕◯◯◯ Very low[e,f,h,n,o]	
Alternate fluency											
1	randomised trials	serious[e]	serious[g]	very serious[f]	very serious[o]	publication bias strongly suspected[h]	-	17		⊕◯◯◯ Very low[e,f,g,h,o]	

N of	Certainty assessment	Effect	Certainty	Importance

studies	Study design	Risk of bias	Inconsistency	Indirectness	Imprecision	Other considerations	N of events	N of individuals	Rate (95% CI)		
Set-shifting accuracy											
1	randomised trials	serious[e]	serious[g]	very serious[f]	very serious[o]	publication bias strongly suspected[h]	-	15		⊕◯◯◯ Very low[e,f,g,h,o]	
Set-shifting response time											
1	randomised trials	serious[e]	serious[g]	very serious[f]	very serious[o]	publication bias strongly suspected[h]	-	15		⊕◯◯◯ Very low[e,f,g,h,o]	
Anxiety											
2	randomised trials	serious[e]	serious[p]	very serious[f]	very serious[q]	publication bias strongly suspected[h]	-	80		⊕◯◯◯ Very low[e,f,h,p,q]	
Theory of Mind											
1	randomised trials	serious[e]	serious[g]	very serious[f]	very serious[g]	publication bias strongly suspected[h]	-	34		⊕◯◯◯ Very low[e,f,g,h]	

Note. (Huguet, A., Hayden, J. A., Stinson, J., McGrath, P. J., Chambers, C. T., Tougas, M. E., & Wozney, L. (2013). Judging the quality of evidence in reviews of prognostic factor research: adapting the GRADE framework. Systematic reviews, 2, 71. https://doi.org/10.1186/2046-4053-2-71).

Explanations

a. Several studies used exploratory predictor analyses, pilot designs, or secondary analyses with potential methodological limitations
b. Predictor effects varied across studies in significance, direction, and statistical approach.
c. Several studies included small sample sizes and limited statistical power
d. Several studies included exploratory analyses and methodological limitations.
e. Prognostic findings were derived from exploratory secondary analyses.
f. Only a small portion of the prognostic factors were considered for this outcome.
g. Evidence was based on a single study with a limited sample size.
h. Publication bias was suspected due to the small number of exploratory studies and limited sample sizes.
i. Publication bias was suspected due to the small number of exploratory studies and limited sample sizes.
j. Prognostic findings were based on exploratory analyses with methodological heterogeneity and secondary analyses.
k. A limited, albeit not small, number of prognostic factors were considered for this outcome.
l. Evidence was based on few studies with small sample sizes.
m. Evidence was based on exploratory analyses from a single small pilot study.
n. Predictor effects varied across studies and phonemic fluency measures.
o. Evidence was based on a single small study with limited statistical power.
p. Predictor effects varied across studies and anxiety outcome measures
q. Evidence was based on few studies with relatively small sample sizes.
